# Characterization of Unfractionated Polysaccharides in Brown Seaweed by Methylation-GC-MS-Based Linkage Analysis

**DOI:** 10.3390/md22100464

**Published:** 2024-10-09

**Authors:** Barinder Bajwa, Xiaohui Xing, Spencer C. Serin, Maria Hayes, Stephanie A. Terry, Robert J. Gruninger, D. Wade Abbott

**Affiliations:** 1 Lethbridge Research and Development Centre, Agriculture and Agri-Food Canada, 5403 1st Avenue South, Lethbridge, AB T1J 4B1, Canada; barinder.bajwa@agr.gc.ca (B.B.); xiaohui.xing@agr.gc.ca (X.X.); stephanie.terry@agr.gc.ca (S.A.T.); robert.gruninger@agr.gc.ca (R.J.G.); 2 Spoitz Enterprises Inc., 215-1610 Pandora Street, Vancouver, BC V5L 1L6, Canada; spencer@spoitzinc.ca; 3 Food BioSciences Department, Teagasc Food Research Centre, Ashtown, D15 KN3K Dublin, Ireland; maria.hayes@teagasc.ie

**Keywords:** methylation analysis, carboxyl reduction, whole food fiber, cell wall, alginate, cellulose, fucoidan, sulfated fucan, laminarin, polysaccharides

## Abstract

This study introduces a novel approach to analyze glycosidic linkages in unfractionated polysaccharides from alcohol-insoluble residues (AIRs) of five brown seaweed species. GC-MS analysis of partially methylated alditol acetates (PMAAs) enables monitoring and comparison of structural variations across different species, harvest years, and tissues with and without blanching treatments. The method detects a wide array of fucose linkages, highlighting the structural diversity in glycosidic linkages and sulfation position in fucose-containing sulfated polysaccharides. Additionally, this technique enhances cellulose quantitation, overcoming the limitations of traditional monosaccharide composition analysis that typically underestimates cellulose abundance due to incomplete hydrolysis of crystalline cellulose. The introduction of a weak methanolysis-sodium borodeuteride reduction pretreatment allows for the detection and quantitation of uronic acid linkages in alginates.

## 1. Introduction

The brown algae (Phaeophyceae) includes approximately 2,000 discovered species, including major marine macrophytes known as brown seaweeds [1]. Brown seaweeds play fundamental roles in coastal marine ecosystems [2] and are harvested as human food, animal feed, and a resource for the extraction of natural products of economic importance [3,4]. Depending on genetic and environmental factors, carbohydrates account for 12–56% of the dry weight of brown seaweeds, consisting of alginic acid, sulfated fucans, laminarin, cellulose, and mannitol [5]. In addition, 1,3-β-d-glucans and mixed-linkage 1,3;1,4-β-d-glucans are also found in some brown seaweed cell walls [6,7]. Alginic acid is a linear polysaccharide composed of 1,4-linked β-d-mannuronic acid (ManA) and α-l-guluronic acid (GulA) [8]. Alginate, the mineral salt form (e.g., sodium salt) of alginic acid, has been widely used in food, medical, pharmaceutical, and cosmetics industries as functional ingredients and biomaterials [9]. The functional properties of alginate have been directly related to the proportions of GulA and ManA, commonly referred to as the G/M ratio [10]. Fucose-containing sulfated polysaccharides (FCSPs) is a collective term used to describe polysaccharides of brown seaweeds incorporating fucose residues and includes sulfated fucans and fucoidans [11]. The backbone of sulfated fucans is composed of fucose linkages that can be further modified with acetyl, sulfate, or monosaccharide residues, such as galactose, fucose, or xylose [12]. Fucoidans encompass a wider spectrum of fucose-rich polysaccharides, containing heterogenous backbones that incorporate uronic acids, galactose, and mannose linkages [13]. FCSPs have many reported bioactivities, including antioxidant, anticancer, anti-inflammatory, antiangiogenic, and antibacterial activities [14,15]. Laminarins are low-molecular-weight storage β-d-glucans synthesized by brown seaweeds, primarily consisting of 3-linked β-d-glucopyranose residues with branching at the *O*-6 position of some residues and occasional 6-linked residues between the 3-linked chains [16]. There are two types of laminarin chains: the G-chain with a free glucose reducing end and the M-chain, where the reducing end is capped by a single d-mannitol residue [17]. Due to the structural diversity and functional variety of polysaccharides in brown seaweeds, it is important to investigate the variations in their chemical compositions among different brown seaweed species across various growth environments, harvest seasons, and different processing treatments, such as blanching.

Methylation-GC-MS analysis is an essential tool for identifying and quantifying glycosidic linkages in polysaccharides, oligosaccharides, and glycoproteins [18,19]. The method involves a multi-step derivatization process that converts monosaccharides, the building units of carbohydrate polymers, into volatile PMAA derivatives, with their anomeric carbon monodeuterium labeled, free hydroxyl groups being methylated, and hydroxyl groups involved in glycosidic linkage formation, sulfate substitution, and sugar ring closure acetylated [20,21]. Whole-cell wall linkage analysis is essential to understand the compositions of unfractionated polysaccharides in biological samples, avoiding the potential loss of specific polysaccharides during fractionation [22]. There have been reports on the glycosidic linkage analysis of unfractionated polysaccharides of various higher plants [23,24,25,26,27], fungi [28,29], and red seaweeds [21]. To date, there has been no glycosidic linkage analysis conducted on unfractionated polysaccharides from brown seaweeds.

Brown seaweed polysaccharides are composed of different types of monosaccharides, which require specific treatments to ensure their quantitative conversion to PMAA derivatives for reliable GC-MS quantitation. Fucoidans contain sulfated residues, which makes them poorly soluble in dimethyl sulfoxide (DMSO) in their natural inorganic salt form for methylation. To enhance solubility in DMSO, they need to be converted to their triethylammonium (TEA) salt form, either through dialysis against a deionized water solution of triethylammonium chloride (TEAC) or by using a cation exchange column [30,31]. The latter method is suitable only for water-soluble polysaccharide fractions and cannot be used on the unfractionated cell wall, as water-insoluble polysaccharides are unable to pass through the column. It was recently reported that ball-milled fine powders of AIRs prepared from red seaweeds formed a homogeneous suspension in DMSO when magnetically stirred overnight at 60 °C [21]. In this process, heated DMSO dissolves non-sulfated polysaccharides, leaving poorly soluble sulfated polysaccharides intact, and the shearing force causes the powder to disintegrate into tiny particles [21]. Cellulose, despite its limited solubility in DMSO due to its highly crystalline structure, can be permethylated using the improved Ciucanu method [32] by forming a suspension in DMSO without visible large pieces. A common method to achieve such a visually homogeneous suspension is by heating ball-milled AIR powder in DMSO with magnetic stirring overnight. This technique was previously proven successful for the permethylation of cellulose in cell walls of higher plants and red seaweeds [21,32]. Satisfactory methylation of a crystalline cellulose standard can be achieved with even one round of methylation [21]. To detect GulA and ManA linkages in alginic acids, carboxyl reduction should be performed before methylation to convert the uronic acids into their 6,6′-dideuterated neutral sugars. Compared to the conventional carbodiimide activation-sodium borodeuteride reduction method [20,33], the weak methanolysis-sodium borodeuteride method (0.5 M methanolic HCl, 80 °C, 20 min) is a quicker, less expensive option that has been recently used for linkage analysis of some cell wall polysaccharide mixtures and purified fractions containing uronic acids [34,35,36,37,38,39]. This method allows larger sample amounts (e.g., 10 mg of AIR) and higher throughput.

Laminarins in brown seaweeds have a historically reported range of degrees of polymerization (DP) from 15 to 30 [17]. The accurate molar masses of laminarins from nine brown seaweed species were recently confirmed by LC-MS to range from 2,000 to 7,000 Da [40]. Therefore, a molecular weight cutoff (MWCO) of 2,000 Da or lower is needed for dialysis after methylation reaction in order to retain laminarin in the dialysis tubing. Unlike in the cell wall analysis of higher plants and fungi, laminarins, instead of starches or glycogen, serve as the storage polysaccharides in brown seaweeds, making amylase treatment of the AIR unnecessary for methylation analysis.

Brown seaweeds display significant variability in the natural abundance and compositions of cell wall polysaccharides depending on genetic and environmental factors [41,42,43]. For example, variations in the contents of alginates and fucoidans were found among different brown seaweed species [41,42,44,45]. For the same species, the composition and detailed structure of polysaccharides vary between harvest seasons and tissue types [41,42,43,45,46,47,48,49]. Structural modification and degradation of the polysaccharides in brown seaweeds can occur during processing treatment [42,50]. Blanching, for example, is an important treatment during food processing to deactivate quality-deteriorating enzymes, decrease microbial load, minimize non-enzymatic browning reactions, enhance dehydration rates and product quality, remove pesticide residues and toxic constituents, expel air entrapped inside plant tissues, and facilitate the peeling of products [51]. Blanching has been widely applied to the processing of seaweeds [52,53,54,55,56] and has been proposed to significantly reduce the iodine content in order to meet food safety standards [57,58]. Therefore, it is imperative to determine the effects of blanching on the polysaccharide compositions of brown seaweeds.

This study aimed to improve the conventional methylation-GC-MS procedure for unfractionated polysaccharide linkage analysis of the brown seaweeds: *Himanthalia elongata* (HE)*, Fucus vesiculosus* (FV), *Alaria marginata* (AM), *Saccharina latissima* (SL), and *Macrocystis tenuifolia* (MT). The variations in linkage compositions of unfractionated polysaccharides were compared across species and between the same species harvested in different years, different tissues, and as a result of blanching.

## 2. Results

### 2.1. Unfractionated Polysaccharide Linkage Compositions of Different Brown Seaweeds

**Figure 1:**
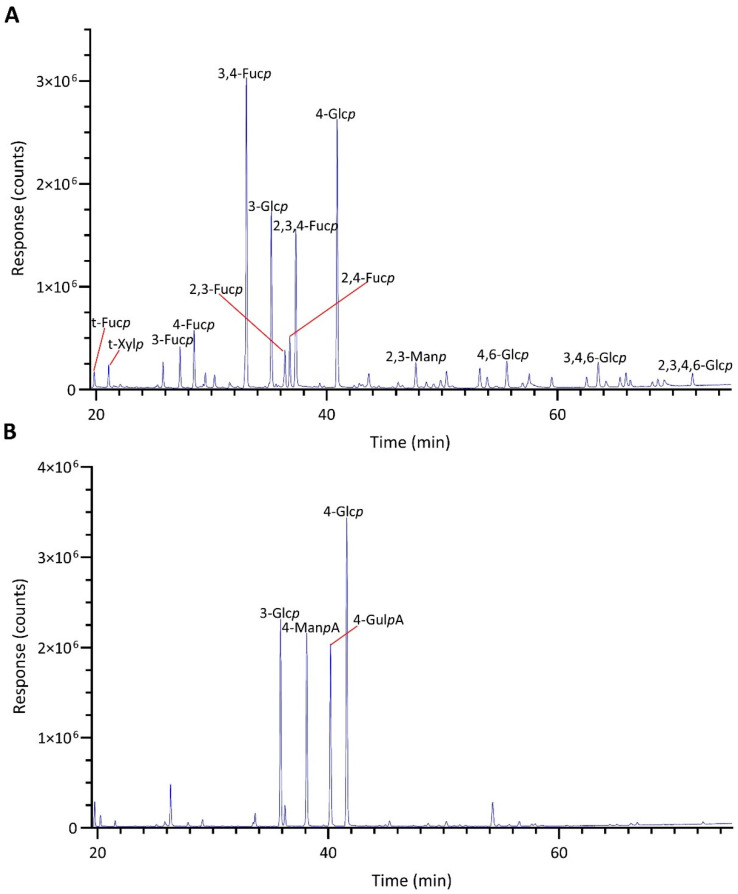
GC-TIC chromatograms of PMAAs from the AIRs of HE: (**A**) without the pretreatment of weak methanolysis-sodium borodeuteride reduction before methylation and (**B**) pretreated with weak methanolysis-sodium borodeuteride reduction before methylation.

**Figure 2:**
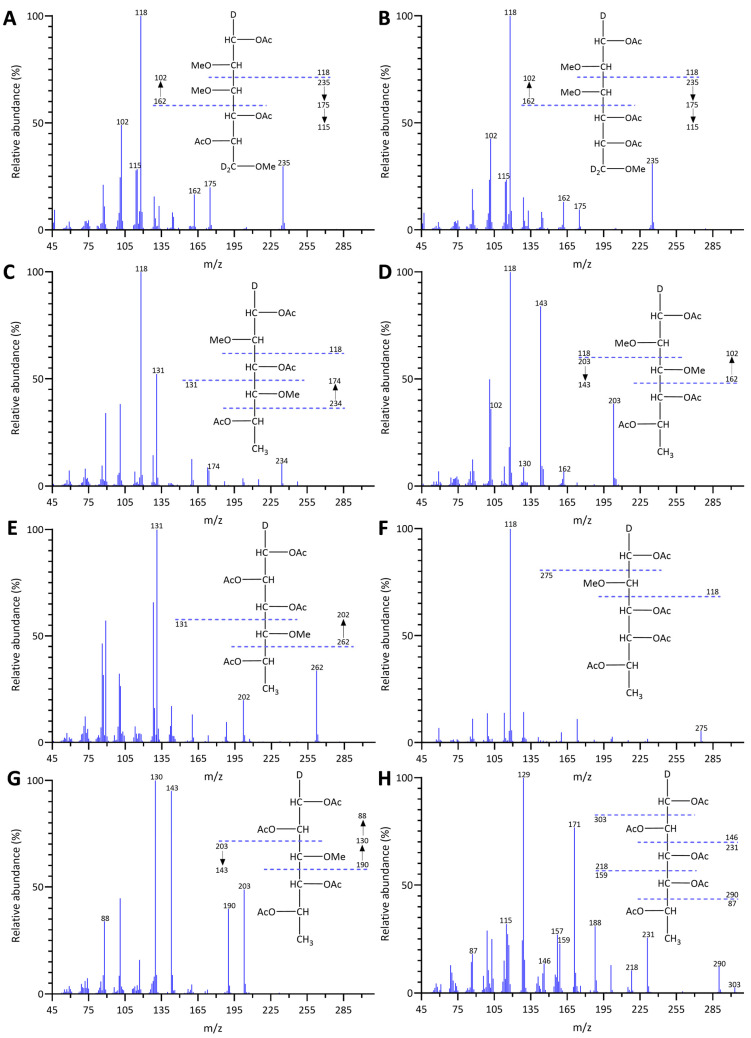
EI-MS spectra and ion fragmentation patterns of PMAAs from (**A**) 4-Gul*p*A, (**B**) 4-Man*p*A, (**C**) 3-Fuc*p*, (**D**) 4-Fuc*p*, (**E**) 2,3-Fuc*p*, (**F**) 3,4-Fuc*p*, (**G**) 2,4-Fuc*p*, and (**H**) 2,3,4-Fuc*p* in HE.

The linkage analysis of unfractionated polysaccharides in five brown seaweed species revealed a total of 72 PMAA signals across all species studied, identified based on their EI-MS fragmentation patterns and retention times, and confirmed by PMAA standards. The GC-TIC chromatogram of PMAAs from HE is presented in Figure 1, and the EI-MS spectra extracted from selected PMAA peaks on the chromatogram are displayed in Figure 2 and supplementary Figure S1. The GC-TIC chromatograms with PMAA peak assignments from example samples of FV, AM, SL, and MT are presented in Figures S2–S5. Example samples presented for all brown seaweed species in this section are whole plants, except for the MT species, whose blade sample was presented. The linkage compositions from these example samples are included in Table 1. Based on these linkage compositions, the relative compositions of monosaccharides were calculated and are displayed in Table S1. The estimated polysaccharide composition for these species is shown in Table S2. The estimation is based on the assignment of linkages to relevant polysaccharides, as shown in Table S3.

#### 2.1.1. Unfractionated Polysaccharide Linkage Compositions of *Himanthalia elongata*

**Figure 3:**
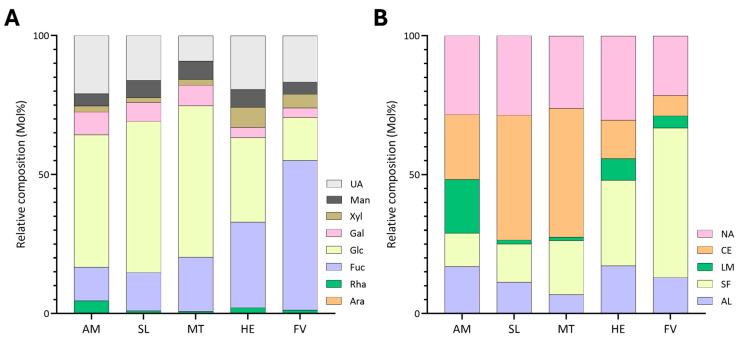
Relative compositions of (**A**) monosaccharides and (**B**) polysaccharides calculated from linkage compositions of AIRs of five brown seaweed species. UA: uronic acids; Man: mannose; Xyl: xylose; Gal: galactose; Glc: glucose; Fuc: fucose; Rha: rhamnose; Ara: arabinose; NA: unassigned linkages; CE: cellulose; LM: laminarin; SF: sulfated fucan. HE was harvested in Q4 of 2020, and FV was harvested in 2020. AM, MT, and SL were harvested in Q2 of 2021. All samples were unblanched.

Glycosidic linkage analysis of HE revealed that the seaweed was primarily composed of 4-Glc*p* at 13.8%, 3,4-Fuc*p* at 12.0%, 4-Gul*p*A at 8.5%, and 4-Man*p*A at 8.3% (Table 1). The predominant polysaccharides were estimated to be sulfated fucans, comprising 30.9% of the total abundance, followed by alginate at 17.1%, cellulose at 13.8%, and, lastly, laminarin at 8.9% (Figure 3B, Table S2). Unassigned linkages accounted for 29.2% (Table S2) and included 2-Xyl*p* at 3.0%, 3,4,6-Man*p* at 1.9%, and 4-Xyl*p* at 1.8% (Table 1). The high proportions of 3,4-Fuc*p* and 2,3,4-Fuc*p* (Table 1) indicated highly branched and sulfated backbones for the sulfated fucans. HE displayed the lowest G/M ratio of 1.0 among all species compared (Table 2). HE also had considerably higher levels of xylose (7.3%) linkages relative to other species (Figure 3A, Table S1).

#### 2.1.2. Unfractionated Polysaccharide Linkage Compositions of *Fucus vesiculosus*

Among the analyzed brown seaweed species, FV exhibited the highest levels of sulfated fucans at 54% (Figure 3B, Table S2), with its structure primarily composed of 2,3,4-Fuc*p* (18.8%), 2,3-Fuc*p* (12.1%), and 2,4-Fuc*p* (6.6%) (Table 1). Notably, high levels of t-Fuc*p* (5.2%) were measured in FV, in contrast to other species (Table 1). Moreover, FV contained the lowest levels of estimated cellulose among all samples at 7.3% of the total polysaccharide composition (Figure 3B, Table S2). The second-most abundant polysaccharide was calculated to be alginate at 12.7%, followed by laminarin at 5.0% (Figure 3B, Table S2). Furthermore, 21.0% of the linkages remained unassigned to any brown seaweed polysaccharide (Figure 3B, Table S2), of which 4-Glc*p*A, t-Xyl*p*, and 2-Xyl*p* were the predominant linkages (Table 1). In contrast to HE, FV contained one of the highest G/M ratios at 4.1 (Table 2), with 4-Gul*p*A and 4-Man*p*A accounting for 10.2% and 2.4% of the total linkages, respectively (Table 1). 

#### 2.1.3. Unfractionated Polysaccharide Linkage Compositions of *Alaria marginata*

AM contained the highest levels of laminarin among all species at 19.9% (Figure 3B, Table S2), with 3-Glc*p* contributing to 18.7% and 3,6-Glc*p* at 0.6% of the total linkage composition (Table 1). The high ratio between 3-Glc*p* and 3,6-Glc*p* suggested that the laminarin backbone contained minimal branching. AM exhibited the lowest levels of sulfated fucan content at 12.1% (Figure 3B, Table S2). The major backbone linkages of the sulfated fucan were 3-Fuc*p*, 3,4-Fuc*p*, and 2,3,4-Fuc*p* (Table 1). Cellulose was the most abundant polysaccharide in AM at 23.3% (Figure 3B, Table S2). Alginate content was measured at 16.8% (Figure 3B, Table S2) and contained a G/M ratio of 3.0 (Table 2). Notably, AM contained considerably higher levels of rhamnose (4.7%) and galactose (8.2%) linkages in contrast to other species (Figure 3A, Table S1). Major linkages contributing to the 27.8% unassigned linkage estimated in Figure 3B and Table S2 included 3,4-Gal*p*, 4-Glc*p*A, and 3-Glc*p*A (Table S1).

#### 2.1.4. Unfractionated Polysaccharide Linkage Compositions of *Saccharina latissima*

The major linkages contributing to the polysaccharide composition of SL were 4-Glc*p* at 45.0%, 4-Gul*p*A at 8.1%, 2,3,4-Fuc*p* at 3.1%, and 4-Man*p*A at 2.8% (Table 1). Due to the prevalence of the 4-Glc*p* linkage, cellulose was estimated to be the most abundant polysaccharide in SL (Figure 3B, Table S2). Sulfated fucans accounted for 12.1% of total polysaccharide composition of SL (Figure 3B, Table S2), with predominant linkages including 2,3,4-Fuc*p*, 3-Fuc*p*, and 3,4-Fuc*p* (Table 1). Alginate was the third-most abundant polysaccharide at 11.2%, with minor levels of laminarin detected (Figure 3B, Table S2). Unassigned linkages accounted for 28.4% (Figure 3B, Table S2) of the polysaccharide composition, including 4-Glc*p*A, 3-Glc*p*A, and 3,4-Gal*p* (Table 1).

#### 2.1.5. Unfractionated Polysaccharide Linkage Compositions of *Macrocystis tenuifolia*

The blades of MT were primarily composed of cellulose (46.5%). The levels of 4-Gul*p*A and 4-Man*p*A accounted for 5.1% and 1.4% of the total linkage composition (Table 1), contributing to the lowest alginate content (6.7%) among all species (Figure 3B and Table S2). Sulfated fucans were estimated to be the second-most abundant polysaccharides in MT at 19.5% (Figure 3B, Table S2), with 2,3,4-Fuc*p*, 3-Fuc*p*, and 2,4-Fuc*p* linkages being the primary contributors (Table 1). The blades of MT contained low levels of laminarin, measured at 1.4% of the total polysaccharide abundance (Figure 3B, Table S2). Furthermore, 25.9% of the linkages remained unassigned to any polysaccharide (Figure 3B, Table S2) and included 3,4-Gal*p*, 3,4,6-Gal*p*, and 4-Glc*p*A.

### 2.2. Variations in Unfractionated Polysaccharide Linkage Compositions Among Brown Seaweed Samples

The linkage compositions of unfractionated polysaccharides were compared among species, for the same species harvested in different years, and between different tissues of the same species with and without blanching treatments, as described in Section 2.2.1, Section 2.2.2 and Section 2.2.3, respectively.

#### 2.2.1. Variations in Unfractionated Polysaccharide Linkage Compositions Among Tissues of *Macrocystis tenuifolia*

**Figure 4:**
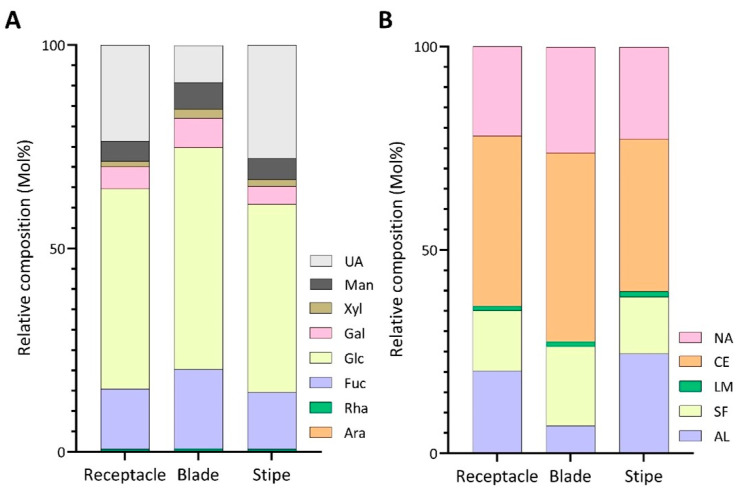
Relative compositions of (**A**) monosaccharides and (**B**) polysaccharides calculated from linkage compositions of AIRs of the receptacle, blade, and stipe of MT harvested in 2021 without blanching. UA: uronic acids; Man: mannose; Xyl: xylose; Gal: galactose; Glc: glucose; Fuc: fucose; Rha: rhamnose; Ara: arabinose; NA: unassigned linkages; CE: cellulose; LM: laminarin; SF: sulfated fucan; AL: alginate.

MT blades contained higher proportions of glucose and fucose (Figure 4A, Table S1) relative to the other tissues, contributing to a higher composition of cellulose (46.5%) and sulfated fucans (19.5%) in the tissue (Figure 4B, Table S2). The sulfated fucan content of the receptacles was 14.8%, followed by stipes at 14.1%, while the cellulose content was estimated to be around 41.9% for the receptacles and 37.6% for the stipes (Figure 4B, Table S2). MT blades exhibited the lowest levels of uronic acid monosaccharides (9.0%) in contrast to the stipe (27.7%) and receptacles (23.6%) (Figure 4A, Table S1). The alginate content reflected this discrepancy, with the blades containing 6.7% alginate, while the stipe and receptacles contained 24.4% and 20.3%, respectively (Figure 4B, Table S2). The stipe and receptacles had an alginate G/M ratio of 1.0 and 1.2, respectively, while the blades contained the highest ratio between the tissues at 3.6 (Table 2).

#### 2.2.2. Annual Variations of Unfractionated Polysaccharide Linkage Compositions of *Alaria marginata* and *Saccharina latissima*

**Figure 5:**
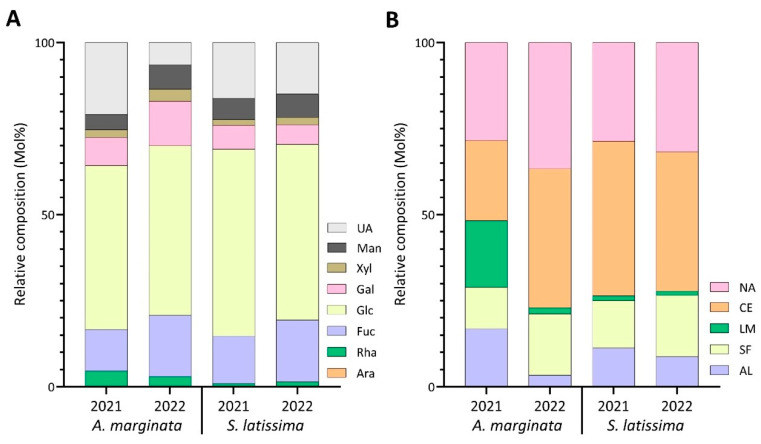
Relative compositions of (**A**) monosaccharides and (**B**) polysaccharides calculated from linkage compositions of AIRs of AM and SL in 2021 and 2022. UA: uronic acids; Man: mannose; Xyl: xylose; Gal: galactose; Glc: glucose; Fuc: fucose; Rha: rhamnose; Ara: arabinose; NA: unassigned linkages; CE: cellulose; LM: laminarin; SF: sulfated fucan; AL: alginate.

**Figure 6:**
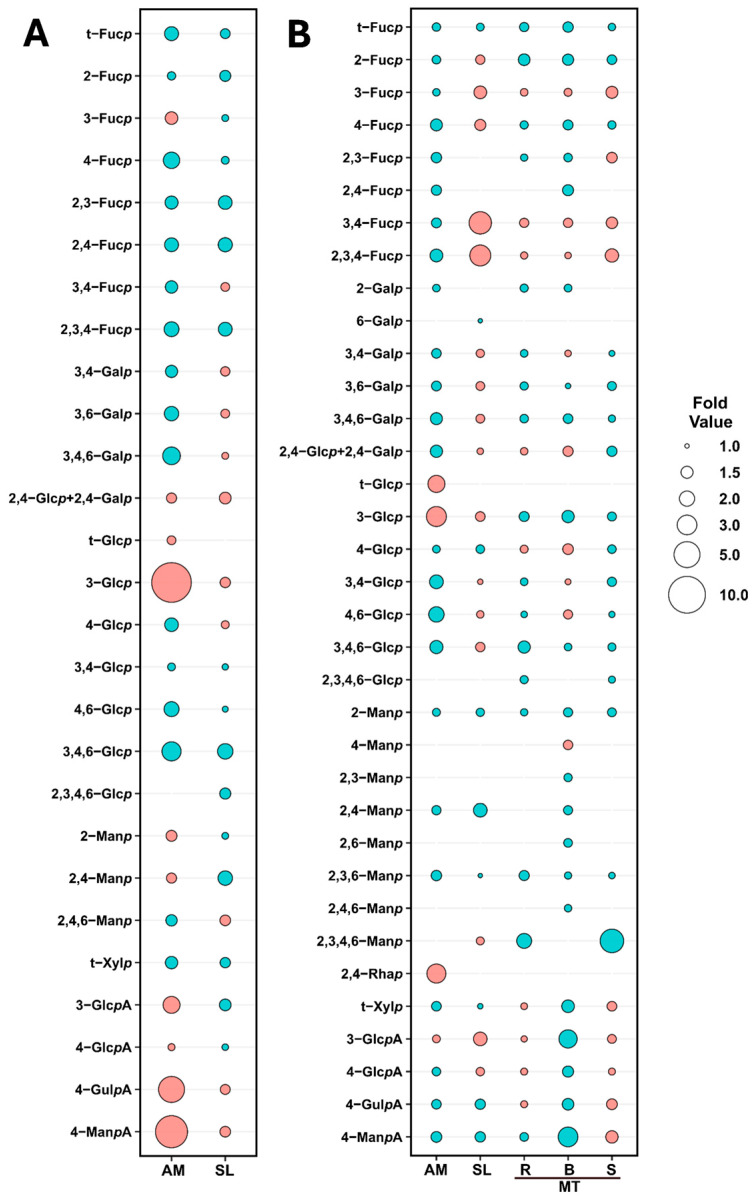
Bubble plots showing fold changes in glycosidic linkages: (**A**) between 2021 and 2022 harvests of AM and SL and (**B**) between blanched and unblanched samples of AM, SL, and the receptacle (R), blade (B), and stipe (S) of MT harvested in 2021. Fold values were calculated as the ratio of the maximum to minimum of each pair of compositions for each linkage, excluding trace-level linkages. Bubble size represents fold value, while bubble color indicates the difference in the pair: coral signifies higher linkage compositions in 2021 compared to 2022 in panel **A** and in unblanched compared to blanched samples in panel **B**, while turquoise indicates the opposite.

Noticeable changes were observed in the linkage composition between the harvest years of AM. Specifically, 3-Glc*p* decreased from 18.7% in 2021 to 1.6% in the 2022 harvest (Table S4) and was reflected in the laminarin content, which reduced from 19.9% to 2.0% (Figure 5B, Table S6). The levels of 4-Man*p*A and 4-Gul*p*A also decreased, going from 4.1% and 12.4% in 2021 to 2.8% and trace levels in 2022 (Figure 6, Table S4). These variations contributed to the lower abundance of alginate between the two harvest years (Figure 5B, Table S6). The drastic reduction in relative alginate abundance between the two harvest years could be attributed to environmental temperature [59], changes in light, or exposure to waves and currents [60]. The relative abundance of cellulose and sulfated fucans increased from 23.3% and 12.1% in 2021 to 40.5% and 17.8% in 2022, respectively (Figure 5B, Table S6). Unassigned linkages increased in 2022 (Figure 5B, Table S6), driven by an increase in mannose, xylose, and galactose linkages (Figure 5A, Table S5). Notably, the 2,4-Rha*p* linkage dropped from 4.3% to trace levels from 2021 to 2022 (Table S4).

The relative abundance of cellulose and alginate was observed to slightly decrease in SL, while sulfated fucans and unassigned linkages increased marginally, and laminarin levels remained relatively stable (Figure 5B, Table S6). SL experienced minor differences in the monosaccharide composition, with the largest change occurring in the fucose content, increasing from 13.8% in 2021 to 17.9% in 2022 (Figure 5A, Table S5). These differences were reflected in the fold change plot in Figure 6, in which the largest change was a two-fold increase in 2,4-Man*p*.

#### 2.2.3. Effects of Blanching Treatments on Unfractionated Polysaccharide Linkage Compositions of *Alaria marginata*, *Saccharina latissima*, and *Macrocystis tenuifolia*

**Figure 7:**
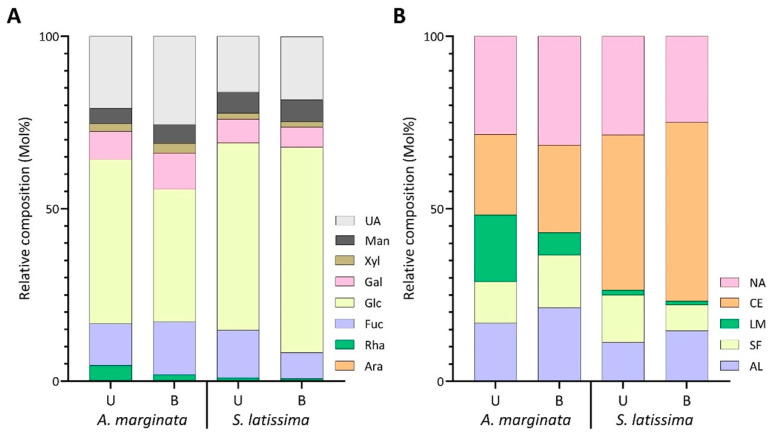
Relative compositions of (**A**) monosaccharides and (**B**) polysaccharides calculated from linkage compositions of AIRs of blanched and unblanched samples of AM and SL harvested in 2021. UA: uronic acids; Man: mannose; Xyl: xylose; Gal: galactose; Glc: glucose; Fuc: fucose; Rha: rhamnose; Ara: arabinose; NA: unassigned linkages; CE: cellulose; LM: laminarin; SF: sulfated fucan; AL: alginate. B and U represent blanched and unblanched samples, respectively.

Blanching treatment in AM caused a significant decrease in estimated laminarin content from 19.3% to 6.4% and an increase in alginate from 16.9% to 21.4%, with minor changes in the relative abundance of sulfated fucans, cellulose, and unassigned linkages (Figure 7B, Table S9). The drop in laminarin and increase in alginate was reflected in a decrease in overall glucose content from 47.7% to 38.5% and, conversely, an increase in uronic acid monosaccharides (Figure 7A, Table S8).

In SL, blanching reduced the sulfated fucan composition from 13.8% to 7.5% (Figure 7B, Table S9). The decrease resulted from reductions to 3,4-Fuc*p* (2.6% to 0.7%) and 2,3,4-Fuc*p* (3.1% to 0.9%), with minor changes in other fucose linkages (Table S7, Figure 6). In contrast, alginate and cellulose saw slight increases, while laminarin levels remained stable between processing methods (Figure 7B, Table S9).

The polysaccharide distribution in MT receptacles remained relatively unchanged during the blanching process. However, there was an increase in unassigned linkages from 21.8% to 25.1%, accompanied by a slight decrease in cellulose from 41.9% to 37.7% (Figure 7B, Table S9).

With the exception of glucose, blanching caused a rise in the relative abundance of all monosaccharides in the blades of MT (Figure 7A, Table S8). In contrast, the estimated glucose composition experienced a large drop upon blanching, going from 54.5% to 43.7% (Figure 7A, Table S8). This increase was also reflected in the relative linkage composition of 4-Glc*p* (Table S7), as well as the cellulose and laminarin polysaccharide distribution (Figure 7B, Table S9). Furthermore, with 4-Man*p*A rising from 1.4% to 4.1% and 4-Gul*p*A increasing from 5.1% to 7.4% (Table S7), estimated alginate levels increased from 6.7% to 11.9% in the blanched blades of MT (Figure 7B, Table S9). The increase in 4-Man*p*A also resulted in a notable decrease in the G/M ratio of alginate from blanched samples, from 3.6 to 1.8 (Table 2).

In the stipes of MT, the drop in sulfated fucan composition (Figure 7B, Table S9) in blanched samples resulted from a reduction in 3-Fuc*p,* 3,4-Fuc*p*, and 2,3,4-Fuc*p* (Figure 6, Table S7) linkages. Meanwhile, cellulose increased from 37.6% to 43.3%, and unassigned linkages increased from 22.5% to 27.5% (Figure 7B, Table S9). The decrease in alginate content in blanched stipes of MT, from 24.4% to 17.0% (Figure 7B, Table S9), was attributed to drops in 4-Man*p*A and 4-Gul*p*A (Figure 6, Table S7). Furthermore, the changes to the alginate linkages resulted in the G/M ratio of alginate to increase from 1.0 to 1.8 in blanched MT stipes (Table 2).

## 3. Discussions

The method reported here enabled the detection of glycosidic linkages from brown seaweed polysaccharides without the need for fractionation. Although fractionation can provide insight into the structure of specific polysaccharides, it can lead to the loss of significant polysaccharides and a reduction in experimental throughput due to the need for derivatizing individual fractions. In contrast, linkage analysis on unfractionated polysaccharides can yield valuable information on the relative compositions of polysaccharides within a sample. Traditional methods typically focus on derivatizing specific fractionated polysaccharides [61,62,63]. However, our methodology consolidated and improved upon previous protocols, successfully enabling the derivatization and detection of linkages in polysaccharides with a wide range of physicochemical properties, including sulfated fucans, crystalline cellulose, water-soluble laminarin, and uronic acid containing alginate. Using this untargeted approach, we identified 72 unique linkages across various seaweed species, highlighting the complexity of the brown seaweed polysaccharide glycome. With these linkages, we were able to estimate the polysaccharide composition of seaweeds and observe differences between harvest year, seaweed tissue, and processing treatments. Glycomics analysis of unfractionated polysaccharides can open avenues for comparative studies to observe changes in polysaccharide composition between various environmental conditions, developmental stages, or treatment groups and can be used to complement analytical techniques, such as HPAEC-PAD, NMR, FT-IR, and glycan arrays [7,20,26]. This information can provide insight into the optimal growth conditions for the extraction of valuable polysaccharides, along with an understanding of the role that linkages and their modifications play in the overall function of polysaccharides.

In our previous study on red seaweeds, we found that ball-milled AIR powder formed a visually homogeneous suspension in DMSO, facilitating the first methylation without needing cation exchange of sulfate counterions from inorganic to TEA salt forms prior to the first round of methylation [21]. Heated DMSO dissolved non-sulfated polysaccharides, dissolving the AIR powder for effective methylation [21]. Applying this method to brown seaweeds, we observed a similar suspension after overnight stirring in heated DMSO. The first methylation product dissolved easily in DMSO post-TEAC dialysis, allowing for an efficient second round of methylation. Even without TEAC dialysis, the twice-methylated sulfated fucans dissolved readily in DMSO, ensuring the successful completion of the third and final methylation round, effectively methylating all free hydroxyl groups.

To detect cell wall polysaccharides of brown seaweeds, weak methanolysis (0.5 M methanolic HCl, 80 °C, 20 min) with sodium borodeuteride was performed. This enabled the detection and comparison of the 4-Gul*p*A and 4-Man*p*A linkages in alginates. The acid-resistant nature of polyuronic acid chain of alginate makes it well-suited for such treatment. Additionally, the 4-Glc*p* linkage from acid-stable crystalline cellulose serves as a reliable internal standard, allowing for the normalization of uronic acid linkages in cell walls with the pretreatment of weak methanolysis-sodium borodeuteride reduction and the neutral sugar linkages in the cell walls without the pretreatment. Notably, we observed the loss of fucose linkages in the GC-TIC chromatograms of PMAAs from the pretreated samples, indicative of methanolysis causing the depolymerization of the sulfated fucan chain to oligosaccharides that were lost during dialysis. SFCPs were previously reported to be susceptible to depolymerization in weak acid conditions [64]. For future research, we suggest collecting the oligosaccharides generated from the weak methanolysis for detailed structural analysis using techniques such as NMR and LC-MS to better understand the structure of the parent polysaccharides [22]. Furthermore, since weak methanolysis is commonly used for carbohydrate desulfation [65,66], conducting methylation-GC-MS analysis on the oligosaccharides and comparing the linkage results with those from untreated samples could potentially reveal the position of sulfation on the fucose sugar ring.

### 3.1. Estimation of FCSPs in Various Brown Seaweed Species

In the current study, many linkages remained unassigned, ranging from 21.4% in FV to 30.3% in HE. Literature suggests that many of these linkages were either branched chains delving off sulfated fucans or involved in the formation of fucoidans, such as fucoglucuronomannans [67] and fucogalactans [68]. These polysaccharides may also be branched with uronic acid, rhamnose, xylose, galactose, and mannose linkages [11]. Despite all the research available characterizing fucoidans, we did not assign linkages for these polysaccharides as it would be difficult to ascertain the origin of these linkages as they can differ among species or could contribute to the formation of glycoproteins. Regardless, we presume that many of the unassigned linkages from Figure 3B originated from fucoidans or side chains of sulfated fucans.

Our findings indicated that the sulfated fucan backbone of HE was primarily composed of 3,4-Fuc*p* and 2,3,4-Fuc*p* linkages (Table 1). These results align well with existing literature, which identified the backbone motif as a chain of 3-linked α-fucose residues with sulfation predominantly at the *O*-4 position [12]. While our study found fucose linkages potentially containing branching, acetyl groups, or sulfate substitutions at the *O*-2 position (Table 1), we were unable to confirm the chemistry of these substitutions. Using ^1^H-NMR analysis of the homofucan fraction, a previous study reported no sulfation at the *O*-2 and *O*-3 positions [12]. Moreover, signals corresponding to acetyl groups were reduced following acid hydrolysis and were theorized to result from natural acetylation [12]. Other species of brown seaweeds, such as *Chorda filum*, have been shown to contain branching and acetyl groups at the *O*-2 position [69], in which case the *O*-2 linkages found in our report could be attributed to branching or natural acetylation. For future research, we recommend employing ^13^C NMR to characterize the purified homofucan and verify the presence of branching or acetyl groups.

HE was also found to contain the highest relative abundance of 2-Xyl*p*, 3-Xyl*p*, and 4-Xyl*p*, along with 3,4,6-Man*p* and 2,3,4,6-Man*p* (Table S4) out of all species. In previous literature, monosaccharide composition analysis of the water-soluble fraction of HE had resulted in the presence of xylose and mannose, which authors speculated were from xylofucoglycouronans or xylomannans [70]. A study characterizing the fucoidan fractions of *Fucus serratus* L. detected NMR signals corresponding to 4-linked Xyl*p*, presumed to be linked to a fucoidan through chains of six xylose residues [71]. Regardless, we presume that xylose and mannose linkages play a key role in the formation of the fucoidan’s structure. Researchers postulate that short-chained hemicelluloses may function as cross-linkers between FCSPs and cellulose microfibrils [12], in which case the presence of mannose and xylose may improve the structural integrity of the brown seaweed cell wall and hinder the ability to extract FCSPs of interest. Moreover, the incorporation of different monosaccharides, such as mannose and xylose, can promote the production of short-chain fatty acids and improve fermentability of polysaccharides [72], as demonstrated in the fraction collected from HE containing cellulose and fucoidans [70].

The structure of the sulfated fucan backbone for FV has been revised multiple times since the polysaccharide was first characterized in 1950 [73]. NMR analysis has determined that the fucan is composed of alternating 3-linked and 4-linked α-fucose residues [74], characteristic for algae from the order Fucales [71,75]. Further structural elucidation through linkage analysis revealed that sulfation occurred at the *O*-3 and *O*-4, *O*-2 and *O*-4, or the *O*-2 positions, while branching could present in any position [76]. In accordance with these previous studies, our results suggested a high degree of sulfation and branching at the *O*-2 position, considering the most abundant linkages were 2,3,4-Fuc*p*, 2,3-Fuc*p*, 2,4-Fuc*p*, and t-Fuc*p* (Table 1).

An acidic fucoidan with a backbone comprising 4-Man*p*, 3-Fuc*p*, 3-Man*p*, and 4,6-Man*p* was identified from FV using methylation analysis and NMR [77]. The heteropolysaccharide was shown to be conducive to the production of short-chain fatty acids while significantly increasing the proliferation of healthy microbiota [77]. While these mannose linkages were detected in our study, the predominant mannose linkages were found to be 2,3-Man*p*, 4-Man*p*, and 2,3,6-Man*p*. These differences may indicate that our fucoidan contained a higher ratio of branching, sulfation, or acetylation or was a fucoidan similar to the one extracted from *Hizikia fusiforme*. The backbone of the fucoidan from *H. fusiforme* is composed of alternating 2-Man*p* and 4-Glc*p*A, with branches occurring at the *O*-3 position and sulfation at the *O*-6 position of mannose [67]. Linkages corresponding to the backbone of *H. fusiforme* were all detected above trace amounts from our dataset (Table 1), indicating the polysaccharides of FV are more complex than originally thought and may contain fucoidans analogous to a seaweed found in east Asia.

The structure of FV fucoidan varies in its sulfation pattern and monosaccharide constituents with the seasons, but it is unclear if these changes are due to the algae’s reproductive stage or seasonal environmental conditions [45,78]. These alterations are known to influence the properties of the polysaccharide, as a higher degree of branching enables the polysaccharide to be easily fermented by bacteria [77], while sulfate residues are thought to improve the response against freezing and shearing [78]. Monitoring the degree of branching or sulfation through methylation analysis could therefore provide information as to the commercial value or resiliency of a seaweed to environmental stressors.

AM, belonging to the family *Alariaceae*, is notable for its production of acetylated and sulfated galactofucans [79,80]. ESI-MS, NMR, and methylation analysis of the galactofucan oligosaccharide fraction in past literature has revealed a backbone of 3-Fuc*p* with sulfation at the *O*-2 and *O*-4 positions [79]. Galactose units are involved in branch formation and are linked at the *O*-2, *O*-4, and *O*-6 positions, with sulfation occurring at the *O*-2, *O*-4, and *O*-6 positions [79]. Methylation of the deacetylated and desulfated galactofucan by the researchers did not reveal any linkages at the *O*-3 of galactose [79]; however, our data suggested that acetylation and/or sulfation might occur at this position due to the observed levels of 3,4-Gal*p*, 3,6-Gal*p*, and 3,4,6-Gal*p* (Table 1). In addition to 3,4-Gal*p*, 3,6-Gal*p*, and 3,4,6-Gal*p* linkages, the high levels of 3-Fuc*p*, 2,3-Fuc*p*, and 2,3,4-Fuc*p* (Table 1) agreed with the branch positions of the galactofucan determined by past literature [79].

The aforementioned study also detected fragments containing branches of HexA and xylose residues, corroborating the presence of 3-Glc*p*A, 4-Glc*p*A, and t-Xyl*p* in our dataset. While trace levels of rhamnose in fucoidan fractions are commonly detected through monosaccharide analysis in research [81,82,83], our AM sample showed high proportions of 2,4-Rha*p*. Previously, one study has reported the presence of sulfated rhamnogalactofucan and sulfated rhamnofucan in brown seaweeds [84]; unrelated, the microalgae *Glossomastix* sp. has been identified to secrete exudates of rhamnofucans [85]. The fractions containing the sulfated rhamnogalactofucan and rhamnofucan from the brown seaweeds *Eclonia cava* and *Sargassum hornery* were found to inhibit the proliferation of human colon cancer and melanoma cells [84]. Meanwhile, the polysaccharide from *Glossomastix* sp. was found to form a fragile hydrogel, which was sensitive to stress, and the authors proposed it to have potential as an anti-setting stabilizer [85]. These findings suggest that AM could produce a rare form of FCSP, which incorporates rhamnose, the structural properties and promising applications of which remain unknown.

FCSPs from SL have been correlated to numerous biological activities as a result of their sulfated structure. Sulfate esters and overall charge of FCSPs are associated with immunomodulation of B lymphocytes, while the presence of uronic acids can enable the sequestration of bile salts and reduced solubility of cholesterol [86]. Fractions consisting of primarily mannogalactofucans and sulfated fucans extracted from SL have also shown to exhibit anti-inflammatory, anticoagulant, antiangiogenic, and antitumor properties [87]. The authors related the high proportions of sulfate residues on the sulfated fucan fraction to the inhibition of tumor growth and heterotypic cell adhesion [87], further supporting the structure–function relationship of FCSPs.

A comprehensive structural analysis of FCSP fractions revealed that the sulfated fucan backbone of SL was comprised of 3-Fuc*p*, with sulfation at the *O*-2 and/or *O*-4 positions, along with terminal fucose units branching from the *O*-2 position [88]. In addition to the sulfated fucan, a fucogalactan containing a core structure of 6-Gal*p* with branches of t-Gal*p* and t-Fuc*p* at the *O*-4 position; a fucoglucuronomannan composed of alternating 4-Glc*p*A and 2-Man*p* have t-Fuc*p* branches at the *O*-3 of mannose; a fucoglucuronan composed of 3-Glc*p*A with t-Fuc*p* branches at the *O*-4 position were found [88]. In accordance with the study [88], our dataset revealed large amounts of 3-Fuc*p* residues, with 2,3,4-Fuc*p* being the most abundant fucose linkage (Table 1) corresponding to the backbone of the highly sulfated fucan. The notable levels of 4-Glc*p*A, 2-Man*p*, and 6-Gal*p* linkages (Table 1) also supported the evidence of fucoglucuronomannans and fucogalactans, although the presence of 2,4-Man*p* might suggest branching or substitution at the *O*-4 position, which was not detected in the compared study [88]. Finally, SL contained the highest relative levels of 3-Glc*p*A among all species, reflected in the proposed fucoglucuronan structure [88]; however, no 3,4-Glc*p*A was detected in our findings, indicative of the *O*-4 fucose substitutions found by the researchers. Since the primary scope of our study was to determine the relative abundance of all polysaccharides found in brown seaweeds, the minor levels of 3,4-Glc*p*A may have been overshadowed by more dominant linkages. Despite this, the majority of linkages detected in existing literature through methylation analysis were also found in our study, highlighting the powerful potential of methylation analysis for examining unfractionated polysaccharides [88,89].

Until recently, *Macrocystis pyrifera* had been used to describe two genetically distinct species of *Macrocystis* located in different hemispheres [90,91]. Thus, the name *Macrocystis tenuifolia* was proposed for all *Macrocystis* sp. found north of Point Conception, California [91]. FCSP fractions obtained from the brown seaweed MT have been extensively studied for their therapeutic potential. Researchers have found that fucoidan from MT displays antioxidant activity [92], significantly delays neutrophil apoptosis, promotes the activation of natural killer cells, increases antibody production, and exhibits other immunomodulatory properties [93], suggesting its potential as a therapeutic agent against infectious diseases and cancer and maintaining the stability of food and medicine.

The fucoidan of MT contains a backbone of either 3-Fuc*p* or alternating 3-Fuc*p* and 4-Xyl*p*, with sulfation at the *O*-3 of fucose, and branches of fucose and 6-Gal*p* [94]. Additionally, mannose, galactose, rhamnose, and glucose monosaccharide constituents are present in the fucoidan fraction [94], consistent with our results (Table 1). It is common to detect multiple FCSPs in brown seaweeds [79,88,95]. The scarcity of 4-Xyl*p* linkages may suggest that the fucoidan with a backbone of 3-Fuc*p* and 4-Xyl*p* might be present in low amounts, while a sulfated fucan with a backbone of 3-Fuc*p* is likely the primary FCSP in MT at the time of harvest. Furthermore, the levels of 4-Glc*p*A and 2-Man*p* linkages could infer the presence of a fucoglucuronomannan, similar to the one found in SL [88].

It is theorized that FCSPs act as cross-linkers between cellulose microfibrils, which form the structural scaffolding in brown algal cell walls [12]. Meanwhile, alginates are believed to interact with polyphenols, creating the matrix in which this cellulose–FCSP scaffold is embedded between [12]. The researchers suggested that there are few covalent interactions between the scaffold and matrix polysaccharides [12], which may explain the observed increase in cellulose and FCSP content, coinciding with a decrease in alginate in the blades, as the seaweed may have altered its polysaccharide composition to enhance structural stability.

### 3.2. Estimation of Cellulose in Various Brown Seaweed Species

Cellulose from seaweeds has garnered increasing commercial interest, as macroalgae contain lower levels of lignin, which can impede the extractability of the polysaccharide [96]. The properties of algae cellulose have shown to have a variety of applications, including a reduction in colon inflammation in mice [97], filtration of particles of 20 nm [98], and production of micro- or nanocrystalline material to be used in the biomedical, food and additive, and electronics industries [99].

This methylation-based method enabled the detection of cellulose alongside other polysaccharides within the unfractionated cell walls and extracellular matrix of brown seaweeds, offering advantages over conventional cell wall monosaccharide analysis, which typically suffers from incomplete glucose release from crystalline cellulose by acid hydrolysis [100]. Moreover, existing literature often prioritizes the characterization and quantification of bioactive polysaccharides from brown algae, such as FCSPs, alginate, and laminarin through fractionation. Consequently, information regarding polysaccharides and molecules associated with cellulose is often lost or overlooked. Conducting methylation analysis also allows for estimating the abundance of cellulose relative to other polysaccharides, facilitating the selection of valuable seaweed species for cellulose production, or lack thereof.

Among the brown seaweed species analyzed, the estimated cellulose content ranged from 7.3% in FV to 46.4% in the blades of MT (Table 1). Similar to other brown seaweed polysaccharides, the diversity in the primary cell wall structures between samples could be attributed to seasonal, species, or geographical variation [42,43,45]. A frequently referenced study on the cellulose content in brown macroalgae found that cellulose comprised approximately 1–8% of the total dry mass, with the content in FV ranging from 1.2–2.8% [101]. The discrepancy between their results and ours may be attributed to the former reporting the mass of cellulose relative to the algae mass after fractionation, while our study reported the relative abundance of cellulose in relation to the entire polysaccharide glycome of the seaweed. Moreover, methylation analysis in our study enabled the verification of cellulose linkages and ability to discern between various polysaccharides present in the sample matrix. In contrast, the cited study [101] did not verify potential contamination or loss of cellulose during chemical fractionation and filtering.

### 3.3. Estimation of Alginate in Various Brown Seaweed Species

Characterization of alginates from various seaweed species by glycosidic linkage analysis indicated that the G/M ratios ranged from 1.0 in HE to 4.2 in FV (Table 2), with the majority of the seaweed species favoring a high G/M ratio. The gelling ability and characteristics of alginate are dictated by their G/M ratio, frequency, and lengths of G, M, and GM blocks and molecular weight of the polysaccharide [102]. Alginates with a high G/M ratio will yield stronger and more rigid gels, while an even distribution of G and M units will confer flexibility, and a low G/M ratio will result in softer gels with more elasticity [62,103,104]. High G/M ratios have been associated with improving the encapsulation of probiotics during digestion of alginate beads by reducing their pore size and increasing the bead strength [105]. Additionally, the application of alginates containing a high G/M ratio may warrant further research as remedial agents against toxic heavy metals [106].

The G/M ratio of alginate obtained from various seaweed species in our dataset was high, in contrast to the literature [107,108,109,110,111]. Along with the extraction parameters used to isolate the alginate [112], growth conditions, seasonal changes [113], and geographical conditions [114] can also influence the characteristics of the polysaccharide. Literature has also demonstrated that the expression of mannuronan C-5 epimerase, the enzyme responsible for the conversion of mannuronic acid into guluronic acid, is higher in winter and early spring when seawater is enriched with nutrients [115]. As AM, SL, and MT were all harvested in spring, it may be possible that the seawater contained high nutrient levels, which in turn promoted the production of G blocks in seaweeds. In addition to monitoring the G/M ratio as a response to seasonal variation, we suggest the analysis of pH, moisture content, viscosity, rigidity, and characterization of alginate by NMR to determine frequency of G, M, and GM blocks for a comprehensive understanding of the mechanical and chemical properties of the alginate, which will in turn influence their industrial applications.

### 3.4. Further Discussions and Future Considerations

As described in Section 2.2, blanching influenced the major linkages of alginates, cellulose, and sulfated fucans across the different species assessed, with variations in minor linkages also noted. Relatively larger standard deviations in the linkage composition of alginates and laminarin from separate experiments conducted with different starting materials of the same sample were observed in the blanched samples compared to the unblanched, making identification of significant changes challenging. This indicates a possible partial loss or relocation of water-soluble polysaccharides in the seaweed tissue during blanching treatment [116], resulting in the uneven distribution of seaweed polysaccharides in the AIR.

It is important to note that the values reported here represent the relative, not absolute, linkage composition by referencing the total detected linkages in the same sample. Changes in one linkage can affect the proportions of the other linkages in the same sample, and these fluctuations can make it difficult to contextualize results. Moreover, higher relative composition of a specific linkage type in one sample does not necessarily indicate a higher absolute level compared to another sample. We recommend combining this method with one that allows for absolute quantitation of monosaccharides to fully understand changes in cell wall polysaccharide composition. For instance, a previous study analyzing duckweed cell walls successfully integrated relative and absolute carbohydrate analysis [117].

Our approach acts as a framework for comparative glycomics of polysaccharides through linkage analysis. We were able to observe differences in the relative linkage composition and estimated polysaccharide composition between tissue, harvest years, species, and processing treatment. The study would have benefited from more technical replicates and a larger sample pool, improving the significance of results through statistical analyses, such as ANOVA [118], and reducing biological variance between samples.

We suggest in future studies the incorporation of a desulfation protocol followed by linkage analysis to confirm the position of sulfate groups [119,120,121]. The hydroxyl groups involved in the formation of glycosidic linkage, sulfation, and sugar ring closure were *O*-acetylated in the final PMAAs. Future research should focus on isolating these polysaccharide components to study their detailed structures.

## 4. Materials and Methods

### 4.1. Brown Seaweed Materials

*Saccharina latissima f. angustissima* (Collins) A.C.Mathieson 2008:17, *Alaria marginata* Postels & Ruprecht 1840:11, and *Macrocystis tenuifolia* Postels & Ruprecht 1840:9 were harvested from the Pacific Northwest in Canada by Cascadia Seaweed Corporation (Sidney, British Columbia, Canada). For seasonal comparisons, *Saccharina latissima* was harvested on April 11, 2021, and May 1, 2022; *Alaria marginata* on April 12, 2021, and May 1, 2022; and *Macrocystis tenuifolia* on May 4, 2021. Once harvested, the seaweeds were rinsed to remove debris. Subsequently, for each seaweed harvested, some underwent blanching in hot water at a solid-to-liquid ratio of 1:10 (w/w) at 95 °C for 2 min with constant agitation, while the others were left without this treatment. The blanched and unblanched samples were air-dried at no higher than 30 °C for 96 h to a degree that the dried seaweeds can be crumbled in hand.

*Fucus vesiculosus* var*. linearis* (Hudson) Kützing 1849 was harvested from the Atlantic coast of Canada on August 14, 2020, and dried by North Atlantic Organics Ltd., Tignish, Prince Edward Island, Canada. *Himanthalia elongata* (Linnaeus) S.F.Gray 1821 was harvested from the Northwest coast of Ireland in November 2020, dried, and coarsely milled to particle size ranging from 250 µm to 1 mm by Sealac Ltd., Sligo, Ireland. The harvest was dried using a low-temperature method that maintained temperatures below 28 °C, as described in the reference [122].

All the dry seaweed samples were shipped in sealed bags at room temperature to the Lethbridge Research and Development Center, Agriculture and Agri-Food Canada. There, the dry seaweed was ball-milled into fine powder using a Retsch Mixer Mill MM 400 ball mill system (Haan, Germany). The resulting dry powders were sealed in 50 mL tubes and stored at −20 °C before analysis.

### 4.2. Preparation of Unfractionated Polysaccharides of Brown Seaweeds

The AIR samples were prepared from brown seaweed using the same procedure as reported previously for red seaweed [21], which was modified based on the literature [23,26,123]. Briefly, the samples were soaked in 40 mL of an 80% ethanol-deionized water solution (*v*/*v*) for varying durations: 8 h, 16 h, and 8 h, with tubes sealed by polytetrafluoroethylene (PTFE)-lined screw caps and rotated on a tube rotator. After each soaking, they were centrifuged at 3000× *g* for 30 min, followed by discarding the supernatant while retaining the residue. The residue underwent three rounds of washing with acetone (40 mL) and three rounds of washing with methanol (40 mL), each for 20 min on the tube rotator. After each washing, the residue was collected by centrifugation at 3000× *g* for 30 min. The final residue was vacuum-dried using a Savant SPD131DDA SpeedVac concentrator and a Savant RVT5105 refrigerated vapor trap (Thermo Fisher Scientific Inc., Waltham, MA, USA). The dried particles were then ground into a fine powder using the ball milling system mentioned in Section 3.1.

### 4.3. Preparation of PMAA Derivatives from Dry AIR Powder of Brown Seaweeds

#### 4.3.1. Permethylation

The ball-milled powder of each brown seaweed AIR was permethylated using the same procedure as reported previously for red seaweed [21]. Briefly, 10 mg of dry ball-milled AIR powder was suspended in 2 mL of DMSO and stirred at 60 °C in a glass tube sealed by PTFE-lined screw cap and with headspace filled with N_2_. Once cooled, around 200 mg of freshly ground NaOH powder was added, and the mixture was stirred for 2 h with tube headspace filled with N_2_. After adding 1.2 mL of methyl iodide, the tube was sealed, wrapped in aluminum foil, and magnetically stirred for 3 h. The mixture was then cooled on ice and partitioned 3 mL of dichloromethane (DCM) with 5 mL of 10% acetic acid (*v*/*v*) in deionized water one time and then with 5 mL of deionized water two times. The upper phases were pooled and evaporated to around half volume, and the lower phase was evaporated to dryness by a flow of N_2_ supplied by a generator. The concentrated upper phase was mixed with the dried lower phase, and the mixture was transferred to dialysis tubing with an MWCO of 2000 Da. After washing the original tube twice with 1 mL of ethanol two times and adding each wash to the dialysis tubing, the sample was dialyzed against running water overnight, against 4 L of 0.1 M TEAC-deionized water solution for 24 h and then against deionized water for 24 h, followed by freeze-drying. The methylation process was repeated two more times for a total of three rounds of methylations, except in the latter two rounds the overnight stirring in DMSO was conducted at room temperature and the 24 h dialysis against 0.1 M TEAC was omitted.

In a separate experiment, uronic acids in each brown seaweed AIR were converted to their 6,6′-dideuterated neutral sugars by sodium borodeuteride reduction of their methyl esters generated by weak methanolysis [34,35,36,37,38,39]. Briefly, approximately 10 mg of ball-milled powder from brown seaweed AIR was magnetically stirred in 2 mL of 0.5 M methanolic hydrochloric acid at 80 °C for 20 min in a glass tube sealed with a PTFE-lined screw cap and with the headspace filled with N_2_. Once cooled, the reaction mixture was evaporated to dryness by N_2_, followed by two rounds of evaporation to dryness in 2 mL of absolute methanol. Around 2 mL of freshly prepared NaBD_4_ in deionized water (10 mg/mL, *w*/*v*) was added, and the mixture was gently stirred at room temperature overnight with the tube sealed by the PTFE-lined cap [26]. After that, glacial acetic acid was added dropwise to the reaction mixture until the cessation of fizzing caused by hydrogen generation, followed by evaporation to dryness under N_2_. The dry sample was then subjected to two rounds of evaporation to dryness in 2 mL of 10% acetic acid in methanol (*v*/*v*), followed by another two rounds of evaporation to dryness in 2 mL of absolute methanol. Following each addition of solvent, the sample was magnetically stirred for 5 min before evaporation by N_2_. Dried samples were then permethylated as described above, with exceptions that the overnight magnetic stirring in DMSO for the first round of methylation was conducted at room temperature instead of 60 °C, the overnight magnetic stirring in DMSO for the second round of methylation was conducted at 60 °C instead of room temperature, and the dialysis against 0.1 M TEAC-deionized water solution was not conducted.

#### 4.3.2. Acid Hydrolysis

Each sample was magnetically stirred in 2 mL of 4 M TFA at 100 °C for 4 h in the glass tube sealed by PTFE-lined screw cap and with headspace filled with N_2_ [21]. After that, the tube was cooled to room temperature, followed by evaporation to dryness by a gentle N_2_ flow produced by an N_2_ generator.

#### 4.3.3. Reduction

Around 2 mL of freshly prepared NaBD_4_ in deionized water (10 mg/mL, *w*/*v*) was added to each tube. The mixture was magnetically stirred overnight at room temperature with the tube sealed by the PTFE-lined cap [26]. After that, the cap was carefully removed, glacial acetic acid was added dropwise until the cessation of the fizzing caused by the release of H_2_, and then the reaction mixture was evaporated to dryness under the N_2_ flow.

#### 4.3.4. Peracetylation and Final Cleanup

Peracetylation was performed according to the literature [31,124]. TFA (0.25 mL) and acetic anhydride (1.25 mL) were added to each tube containing the dry sample, followed by magnetically stirring the mixture at 60 °C for 1 h, with the tube capped and headspace filled with N_2_. Once cooled, the sample was evaporated to dryness, followed by partitioning DCM (3 mL) with saturated NaHCO_3_ deionized water solution (3 mL) two times and then with deionized water (3 mL) three times. During partitioning with the NaHCO_3_ solution, the tube was rigorously magnetically stirred for 10 min with cap loosely on to release the CO_2_ generated. During partitioning with deionized water, the tube was sealed and rotated on a tube rotator for 20 min. After each partitioning, the upper phase was carefully removed and discarded, and the lower phase was retained for the next round of partitioning. After the final partitioning, the DCM phase was passed through a glass wool-clogged Pasteur pipette loaded with anhydrous Na_2_SO_4_ powder [125]. The DCM solution was evaporated to dryness under N_2_. The sample was then redissolved in ethyl acetate and transferred to a GC vial for GC-MS analysis.

For each brown seaweed AIR, two separate experiments were performed as described in Section 4.3.1, Section 4.3.2, Section 4.3.3 and Section 4.3.4, except that three separate experiments were conducted to the AIRs of *Saccharina latissima* and *Alaria marginata* harvested in 2022.

### 4.4. GC-MS Analysis of PMAAs Prepared from Brown Seaweed AIRs

All PMAAs were analyzed using an Agilent 7890A-5977B GC-MS system (Agilent Technologies, Santa Clara, CA, USA) coupled to a medium-polarity Supelco SP-2380 column (60 m × 0.25 mm × 0.2 µm; Sigma-Aldrich, St. Louis, MI, USA) under a consistent flow of helium at 0.8 mL/min. The inlet temperature was 250 °C. Each sample solution (1 µL) was auto-injected to the system with a 10:1 split ratio. A solvent delay of 12 min was used. The oven temperature was programed to start at a temperature of 120 °C (hold 1 min), followed by increases at 3 °C/min to 200 °C (hold 50 min) and then to 250 °C (hold 20 min). The transfer line temperature was set to 280 °C. Mass spectra were recorded at an ionization energy of 70 eV, an ion source temperature of 230°С, a quadrupole temperature of 150 °C, and scanning in the range of *m*/*z* 45 to 350 at a scan speed of 4.59 scans per second. Agilent OpenLab CDS software version 2.5 (Agilent Technologies, Santa Clara, CA, USA) was used for data acquisition, peak assignment, and peak integration. Identification of the PMAAs was based on the comparison of retention times and EI-MS spectra of the PMAAs with those of reference derivatives generated from polysaccharide standards and methyl glycosides, as described in our previous report [21] and by referring to the literature [18].

### 4.5. Statistical Analysis

Relative molar linkage compositions of the PMAAs were calculated based on the TIC chromatogram, using the concept that the quantity of a PMAA is proportional to the ratio of its TIC peak area to its molecular mass, as per the published protocol [20], with the 4-Glc*p* peak used to normalize the abundances of neutral sugar PMAAs in the sample without weak methanolysis-NaBD_4_ reduction pretreatment and the abundances of PMAAs from uronic acid linkages in the same sample with the pretreatment. The GraphPad Prism software version 8.0.2 (GraphPad Software Inc., California, USA) was used to generate all figures related to the linkage analysis results, except that the bubble plot figures of the fold changes were produced using R-Studio (Posit PBC, Boston, MA, USA) with the ggplot2 [126] and reshape2 [127] packages. Complete R codes for making the figures are available in the supplementary document of our previous study [21]. Fold change analysis was performed comparing AM and SL samples harvested in 2021 to their respective counterparts from 2022. Additionally, unblanched AM, SL, and MT samples were compared to their blanched counterparts. Fold change values were calculated as the ratio of the maximum to minimum composition for each linkage, excluding trace-level linkages.

## 5. Conclusions

The method used in this work enables the determination of various polysaccharide structures in brown seaweeds and the comparison of variations in linkage compositions of unfractionated polysaccharides among species, for the same species harvested in different years, and between different tissues of the same species. The diverse types of fucose linkages observed demonstrate the structural variety in terms of the positions of glycosidic linkage formation and sulfate substitution in FCSPs. This method offers advantages in cellulose quantitation compared to conventional monosaccharide composition analysis that often underestimates cellulose due to the incomplete hydrolysis of crystalline cellulose. Uronic acid linkages from alginates can be detected and quantified by adding a weak methanolysis-NaBD_4_ reduction pretreatment prior to methylation-GC-MS analysis. Given these advantages, this method has significant potential for comparing different disease states in seaweed to investigate how diseases impact polysaccharide composition or to monitor optimal harvesting conditions for commercially valuable polysaccharides. Additionally, it could be applied to select seaweed species based on their polysaccharide profiles relative to other species. This selection process could help optimize the subsequent extraction and purification procedures, thereby minimizing the expenses associated with scaled-up methods required for obtaining the targeted polysaccharides.

## Figures and Tables

**Table 1:** Relative Glycosidic Linkage Composition (Mol%) of Unfractionated Polysaccharides of AM, SL, FV, and HE and the Receptacle, Blade, and Stipe of MT.

Linkage	HE	FV	AM	SL	MT		
					Receptacle	Blade	Stipe
t-Fuc*p*	0.8 ± 0.1	5.2 ± 0.6	1.7 ± 0.1	1.8 ± 0.7	1.2 ± 0.1	1.6 ± 0.2	1.2 ± 0.2
2-Fuc*p*	0.8 ± 0.1	3.5 ± 0.2	1.0 ± 0.1	0.8 ± 0.0	0.7 ± 0.0	1.1 ± 0.1	0.8 ± 0.2
3-Fuc*p*	2.1 ± 0.0	1.8 ± 0.1	2.8 ± 0.1	2.7 ± 0.6	2.7 ± 0.2	3.0 ± 0.5	2.6 ± 0.7
4-Fuc*p*	2.7 ± 0.0	3.7 ± 0.2	1.0 ± 0.2	1.6 ± 0.1	1.0 ± 0.1	1.4 ± 0.2	0.9 ± 0.0
2,3-Fuc*p*	2.9 ± 0.9	12.1 ± 1.5	1.0 ± 0.1	0.8 ± 0.6	0.7 ± 0.0	1.5 ± 0.0	0.7 ± 0.1
2,4-Fuc*p*	2.7 ± 0.0	6.6 ± 0.9	0.6 ± 0.1	Trace	Trace	0.7 ± 0.0	Trace
3,4-Fuc*p*	12.0 ± 0.4	2.2 ± 0.0	2.3 ± 0.2	2.6 ± 1.9	2.2 ± 0.6	2.5 ± 0.6	2.5 ± 0.2
2,3,4-Fuc*p*	6.9 ± 0.6	18.8 ± 0.8	1.7 ± 0.2	3.1 ± 0.1	5.8 ± 0.1	7.7 ± 0.1	4.9 ± 0.8
2-Gal*p*	Trace	Trace	1.0 ± 0.0	0.7 ± 0.1	0.6 ± 0.0	0.6 ± 0.0	Trace
3,4-Gal*p*	Trace	Trace	3.3 ± 0.4	1.9 ± 0.2	1.9 ± 0.1	2.6 ± 0.2	1.5 ± 0.2
3,6-Gal*p*	1.0 ± 0.1	Trace	0.6 ± 0.0	1.2 ± 0.5	0.9 ± 0.0	1.4 ± 0.2	0.6 ± 0.1
3,4,6-Gal*p*	0.6 ± 0.0	Trace	1.3 ± 0.1	1.8 ± 0.7	1.3 ± 0.2	1.8 ± 0.2	1.1 ± 0.1
2,4-Glc*p* + 2,4-Gal*p*	1.3 ± 0.3	0.6 ± 0.0	0.8 ± 0.0	1.8 ± 0.7	1.7 ± 0.2	1.5 ± 0.1	1.8 ± 0.3
t-Glc*p*	1.2 ± 0.1	Trace	1.4 ± 0.1	Trace	Trace	Trace	Trace
3-Glc*p*	6.6 ± 0.8	3.9 ± 0.6	18.7 ± 0.2	1.2 ± 0.1	0.9 ± 0.2	1.1 ± 0.0	1.2 ± 0.2
4-Glc*p*	13.8 ± 0.4	7.3 ± 1.5	23.3 ± 0.3	45.0 ± 3.4	41.9 ± 5.0	46.5 ± 1.1	37.6 ± 2.0
3,4-Glc*p*	1.5 ± 0.0	Trace	0.7 ± 0.1	1.5 ± 0.1	1.4 ± 0.0	1.3 ± 0.1	1.7 ± 0.4
3,6-Glc*p*	1.2 ± 0.2	0.6 ± 0.0	0.6 ± 0.1	Trace	Trace	Trace	Trace
4,6-Glc*p*	1.6 ± 0.0	Trace	Trace	1.4 ± 0.1	1.3 ± 0.0	1.4 ± 0.0	1.6 ± 0.6
3,4,6-Glc*p*	1.3 ± 0.3	0.8 ± 0.4	0.7 ± 0.3	1.2 ± 0.6	0.6 ± 0.0	1.5 ± 0.1	0.7 ± 0.1
2,3,4,6-Glc*p*	0.7 ± 0.0	Trace	Trace	1.0 ± 0.9	0.6 ± 0.3	Trace	0.8 ± 0.3
2-Man*p*	Trace	Trace	1.0 ± 0.1	1.2 ± 0.1	1.0 ± 0.0	0.9 ± 0.1	1.3 ± 0.5
4-Man*p*	Trace	0.6 ± 0.0	Trace	Trace	Trace	0.7 ± 0.0	Trace
2,3-Man*p*	0.8 ± 0.1	0.9 ± 0.1	Trace	Trace	Trace	0.7 ± 0.1	Trace
2,4-Man*p*	Trace	Trace	0.8 ± 0.0	0.6 ± 0.7	Trace	Trace	Trace
2,6-Man*p*	Trace	Trace	Trace	Trace	Trace	0.7 ± 0.1	Trace
2,3,6-Man*p*	Trace	0.6 ± 0.0	0.6 ± 0.1	1.0 ± 0.6	0.6 ± 0.0	0.9 ± 0.0	0.7 ± 0.2
2,4,6-Man*p*	Trace	Trace	Trace	Trace	Trace	0.9 ± 0.1	Trace
3,4,6-Man*p*	1.9 ± 1.7	Trace	Trace	Trace	Trace	Trace	Trace
2,3,4,6-Man*p*	1.4 ± 0.8	0.6 ± 0.0	Trace	Trace	0.8 ± 0.4	Trace	0.6 ± 0.4
2,4-Rha*p*	1.5 ± 0.2	0.9 ± 0.1	4.3 ± 0.1	Trace	Trace	Trace	Trace
t-Xyl*p*	1.4 ± 0.1	2.2 ± 0.1	1.4 ± 0.2	1.0 ± 0.1	0.8 ± 0.1	1.5 ± 0.2	0.9 ± 0.1
2-Xyl*p*	3.0 ± 0.3	1.6 ± 0.0	Trace	Trace	Trace	Trace	Trace
3-Xyl*p*	0.8 ± 0.2	Trace	Trace	Trace	Trace	Trace	Trace
4-Xyl*p*	1.8 ± 0.1	0.7 ± 0.1	Trace	Trace	Trace	Trace	Trace
3-Glc*p*A	Trace	0.8 ± 0.0	1.8 ± 0.3	2.1 ± 0.1	0.8 ± 0.1	0.6 ± 0.2	0.8 ± 0.0
4-Glc*p*A	1.4 ± 0.0	2.3 ± 0.4	2.0 ± 0.5	2.5 ± 0.3	2.3 ± 0.2	1.6 ± 0.1	2.4 ± 0.1
4-Gul*p*A	8.5 ± 0.1	10.2 ± 3.1	12.4 ± 0.7	8.1 ± 0.3	10.8 ± 2.9	5.1 ± 0.7	12.1 ± 2.2
4-Man*p*A	8.3 ± 0.6	2.4 ± 0.5	4.1 ± 0.2	2.8 ± 0.1	8.9 ± 2.6	1.4 ± 0.2	11.9 ± 2.8

Note: Trace means Mol% < 0.5%. AM, SL, and MT were harvested in 2021. FV and HE were harvested in 2020. All samples were unblanched. Two separate experiments were conducted on each sample. The following linkages were found in trace amounts for all samples: 3-Ara*f*, 5-Ara*f*, t-Gal*p*, 4-Gal*p*, 6-Gal*p*, 4,6-Gal*p*, 2,3,6-Gal*p*, 2,4,6-Gal*p*, 2,3,4,6-Gal*p*, 6-Glc*p*, 2,3-Glc*p*, 2,3,6-Glc*p*, 2,4,6-Glc*p*, t-Man*p*, 3-Man*p*, 3,4-Man*p*, 3,6-Man*p*, 4,6-Man*p*, t-Rha*p*, 2-Rha*p*, 3-Rha*p*, 4-Rha*p*, 2,3-Rha*p*, 3,4-Rha*p*, 2,3,4-Rha*p*, 2,4-Xyl*p*, 3,4-Xyl*p*, 2,3,4-Xyl*p*, t-Gal*p*A, 2,4-Glc*p*A+2,4-Gal*p*A, t-Glc*p*A, t-Gul*p*A, and t-Man*p*A.

**Table 2:** Ratio of GulA to ManA (G/M) of Alginates in AIRs Prepared from Selected Brown Seaweed Samples.

AM				SL		HE	FV	MP					
2021		2022		2021				Blade		Stipe		Receptacle	
U	B	U	B	U	B	U	U	U	B	U	B	U	B
3.0 ± 0.3	2.7 ± 0.7	4.3 ± 1.0	3.6 ± 0.6	2.8 ± 0.1	2.9 ± 1.2	1.0 ± 0.1	4.1 ± 0.4	3.6 ± 0.1	1.8 ± 0.4	1.0 ± 0.1	1.8 ± 1.1	1.2 ± 0.0	1.0 ± 0.2

Note: U and B denote unblanched and blanched samples, respectively. The 2021 and 2022 harvests were compared for AM. Blade, stipe, and receptacle were compared for MT harvested in 2021. SL was harvested in 2021. HE and FV were harvested in 2020. The G/M was calculated as the sum of linkage compositions of 4-Gul*p*A and t-Gul*p*A divided by the sum of linkage compositions of 4-Man*p*A and t-Man*p*A. Two separate experiments were conducted for each sample except for the 2022 harvest of AM, where three separate experiments were conducted.

## Data Availability

The data that support the findings of this study are available on request.

## Supplementary Materials

The supporting information can be downloaded at: https://www.mdpi.com/article/10.3390/md22100464/s1. The supporting documents include supplemental flow diagrams showing the steps of the preparation of PMAAs (Schemes S1–S2), supplemental EI-MS spectra of example PMAAs (Figure S1), supplemental GC-TIC chromatograms of PMAAs (Figures S2–S18), supplemental tables for cell wall analysis (Tables S1–S9), supplemental table for PMAA abbreviations (Table S10), and a supplemental diagram of PMAAs generated from seaweed polysaccharides (Scheme S3).
