# Supplementary material for: Characterization of Unfractionated Polysaccharides in Brown Seaweed by Methylation-GC-MS-Based Linkage Analysis
Source: Mar Drugs. 2024 Oct 9;22(10):464. doi: 10.3390/md22100464 (PMC11509683; doi:10.3390/md22100464)
Supplement: Supplementary file 1 [file marinedrugs-22-00464-s001.zip › marinedrugs-3227720-supplementary.pdf]

Supporting documents

Table of contents

Contents	Page
Supplimental flow diagrams showing the steps of the preparation of PMAAs (**Schemes S1-S2**)	2-3
Supplemental EI-MS spectra of example PMAAs **(Figure S1)**	4
Supplemental GC-TIC chromatograms of PMAAs (**Figures S2-S18**)	5-21
Supplemental tables for cell wall analysis (**Tables S1-S9**)	22-33
Supplemental table for PMAA abbreviations (**Table S10)**	34-36
Supplemental diagram of PMMAs generated from seaweed polysaccharides (**Scheme S3**)	37

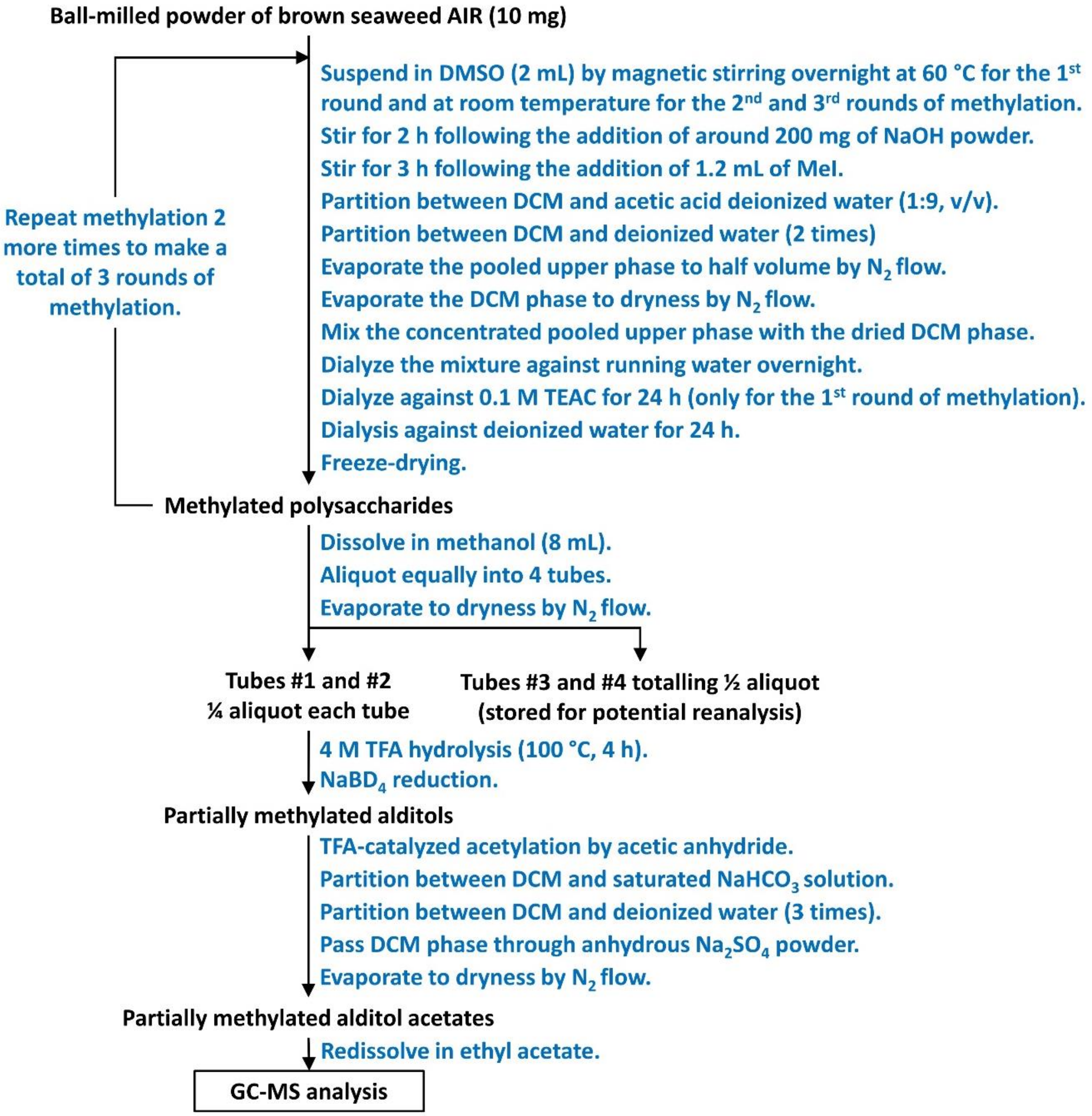

Scheme S1. Flow diagram showing the steps of the preparation of PMAAs from unfractionated polysaccharides in brown seaweed AIR without the pretreeamnt of weak methanolysis-sodium borodeuteride reduction.

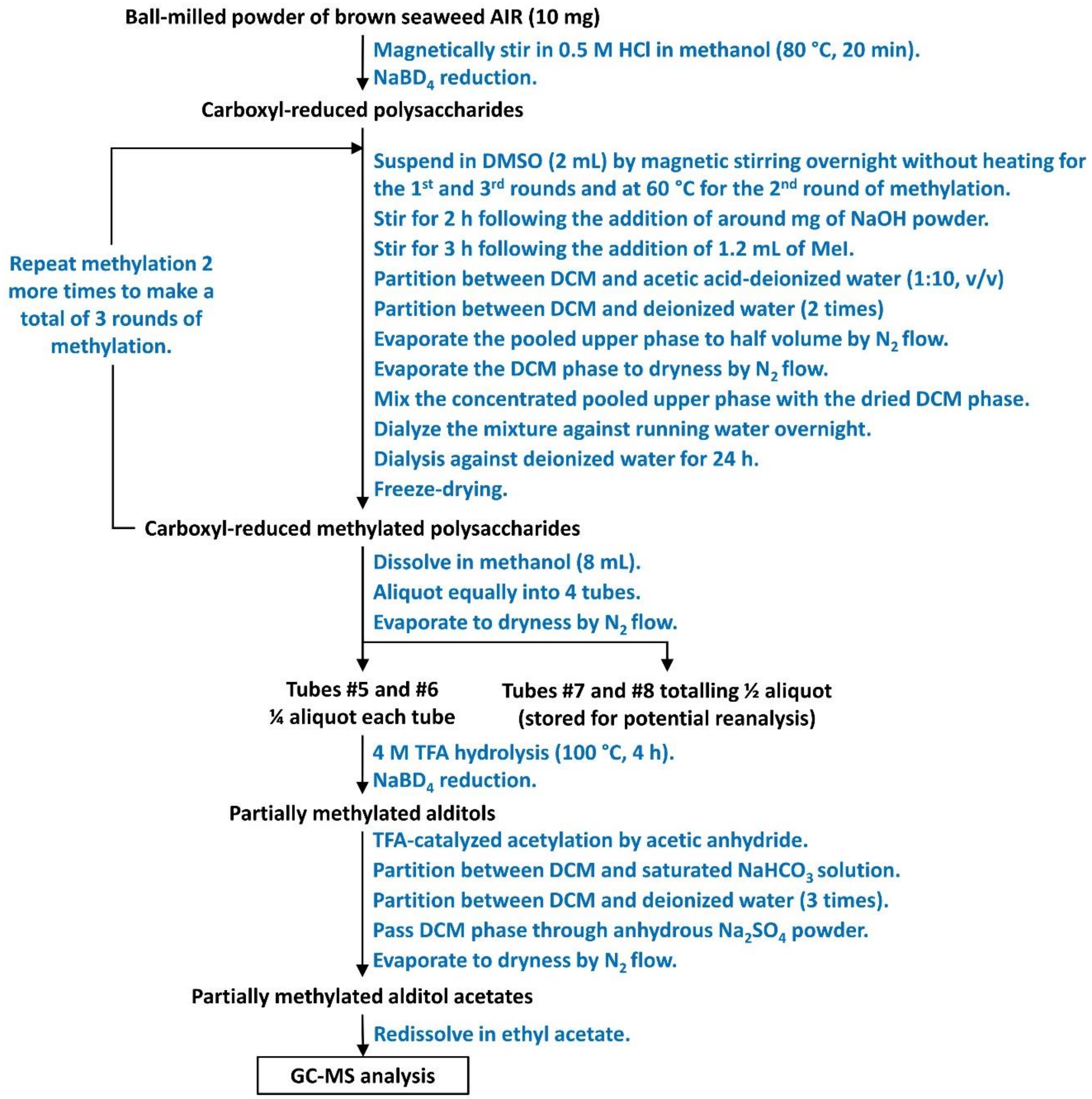

Scheme S2. Flow diagram showing the steps of the preparation of PMAAs from unfractionated polysaccharides in brown seaweed AIR with the pretreatment of weak methanolysis-sodium borodeuteride reduction.

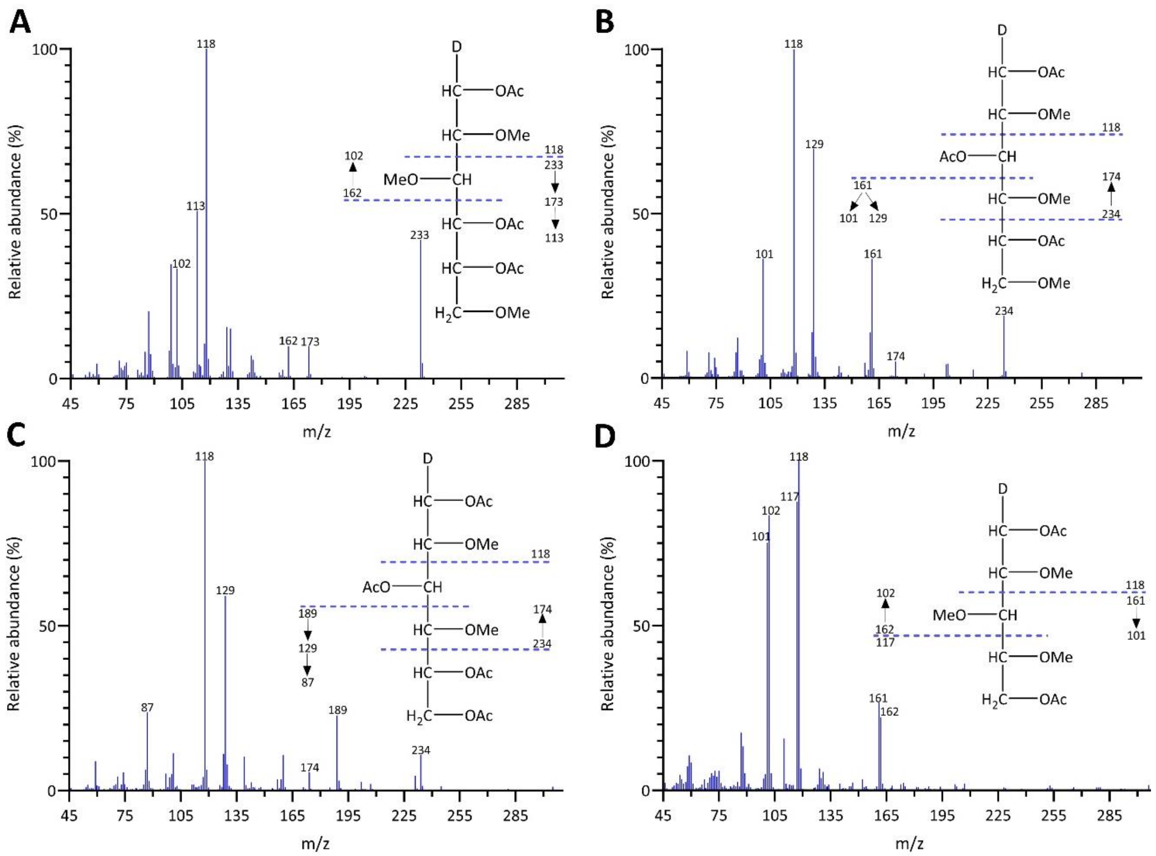

Figure S1. EI-MS spectra and ion fragmentation patterns of PMAAs from (**A**) 4-Glc*p*, (**B**) 3-Glc*p*, (**C**) 3,6-Glc*p*, and (**D**) t-Xyl*p* in *Himanthalia elongata* (HE).

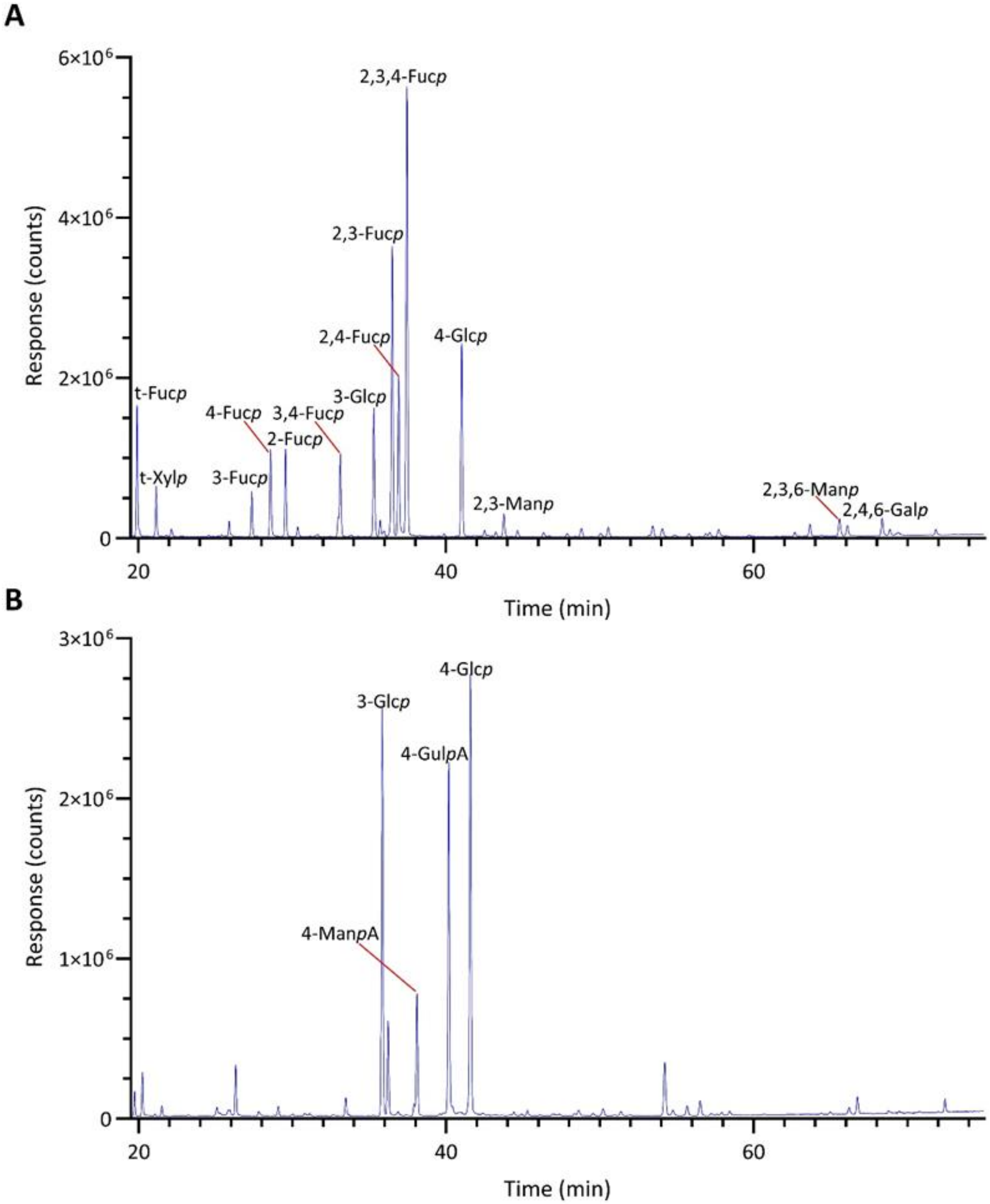

Figure S2. GC-TIC chromatograms of PMAAs from the AIRs of unblanched FV: (**A**) without the pretreatment of weak methanolysis-sodium borodeuteride reduction before methylation, and (**B**) pretreated with weak methanolysis-sodium borodeuteride reduction before methylation.

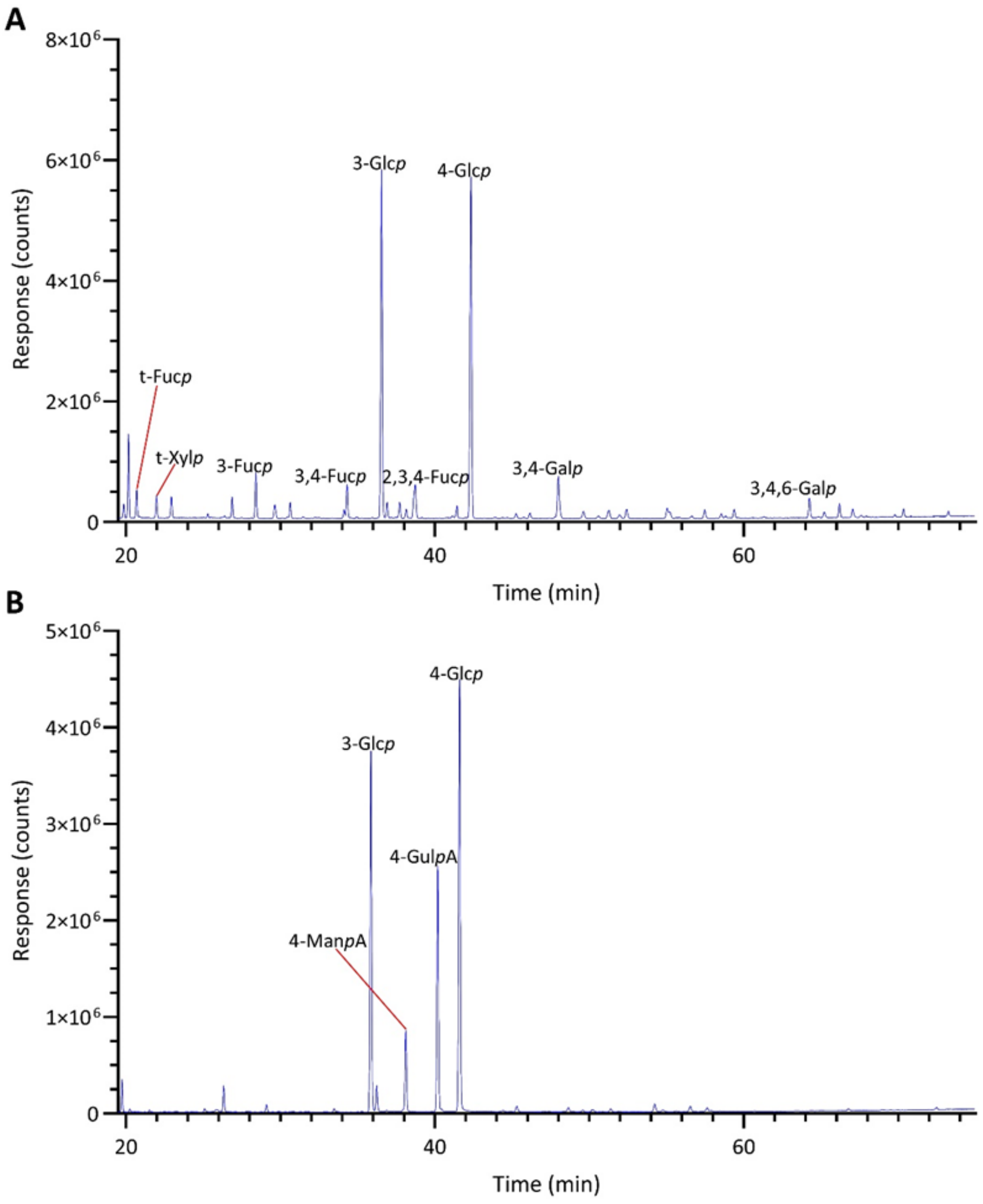

Figure S3. GC-TIC chromatograms of PMAAs from the AIRs of unblanched AM harvested in 2022: (**A**) without the pretreatment of weak methanolysis-sodium borodeuteride reduction before methylation, and (**B**) pretreated with weak methanolysis-sodium borodeuteride reduction before methylation.

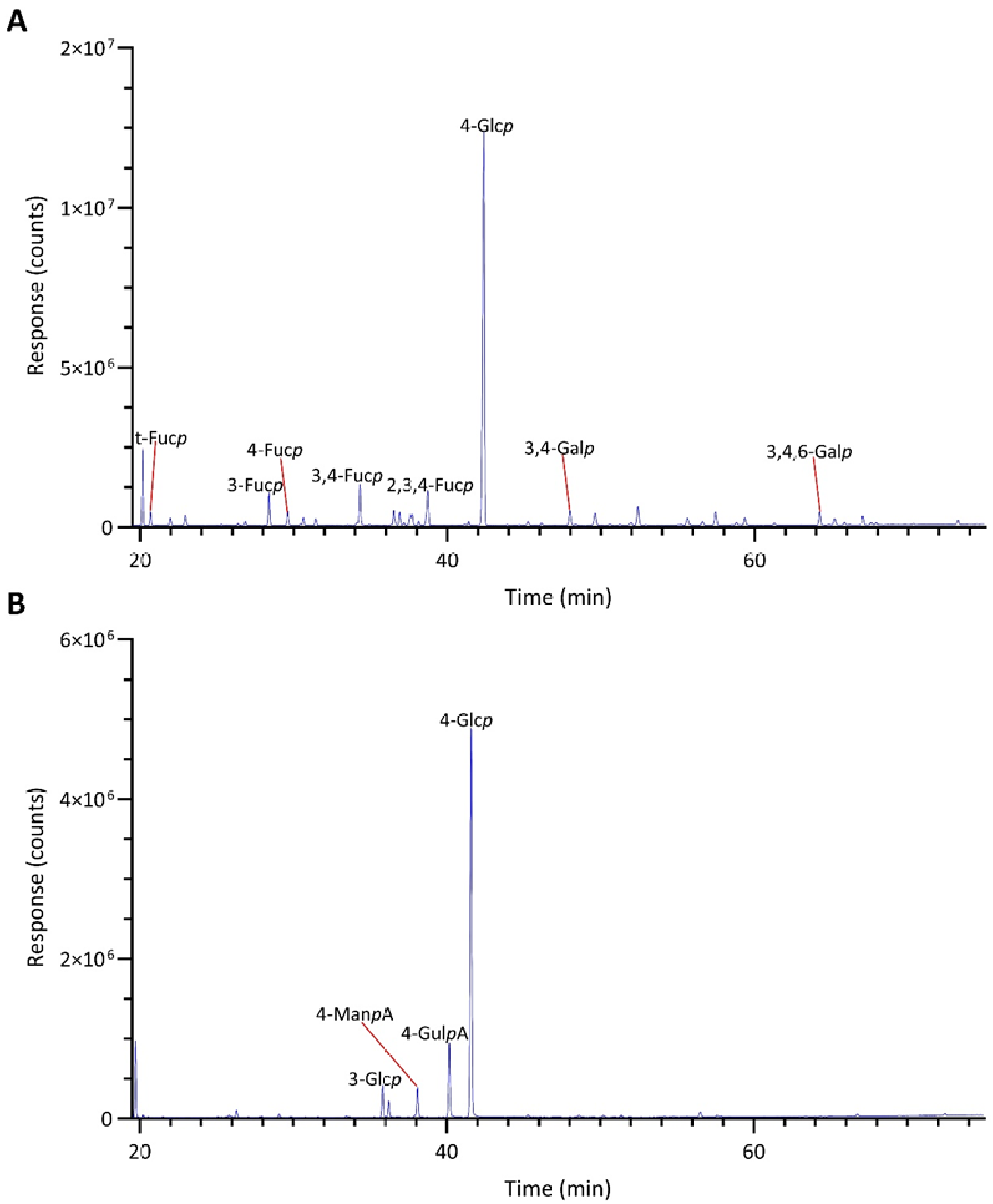

Figure S4. GC-TIC chromatograms of PMAAs from the AIRs of unblanched SL harvested in 2022: (**A**) without the pretreatment of weak methanolysis-sodium borodeuteride reduction before methylation, and (**B**) pretreated with weak methanolysis-sodium borodeuteride reduction before methylation.

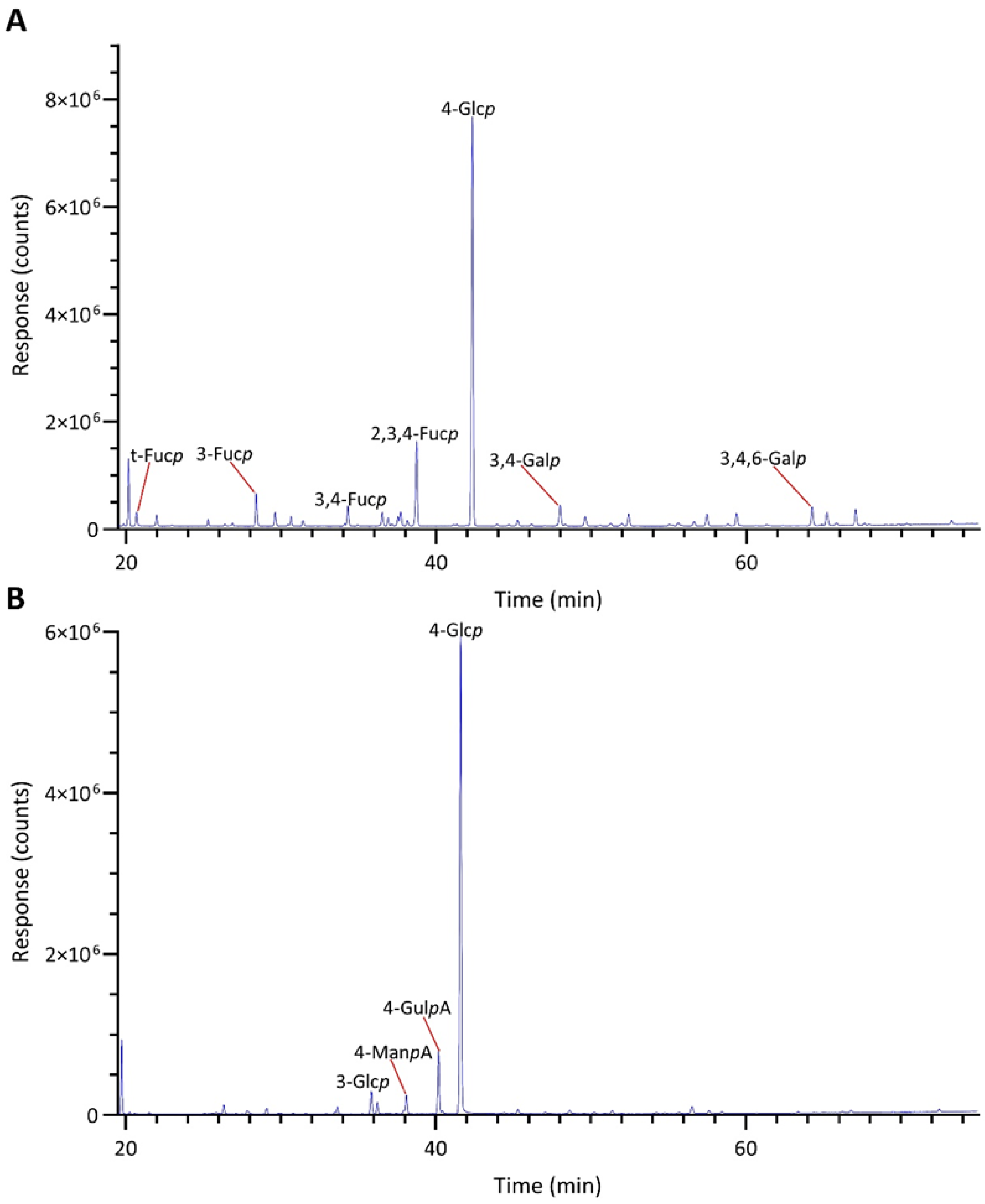

Figure S5. GC-TIC chromatograms of PMAAs from the AIRs of unblanched MT blades: (**A**) without the pretreatment of weak methanolysis-sodium borodeuteride reduction before methylation, and (**B**) pretreated with weak methanolysis-sodium borodeuteride reduction before methylation.

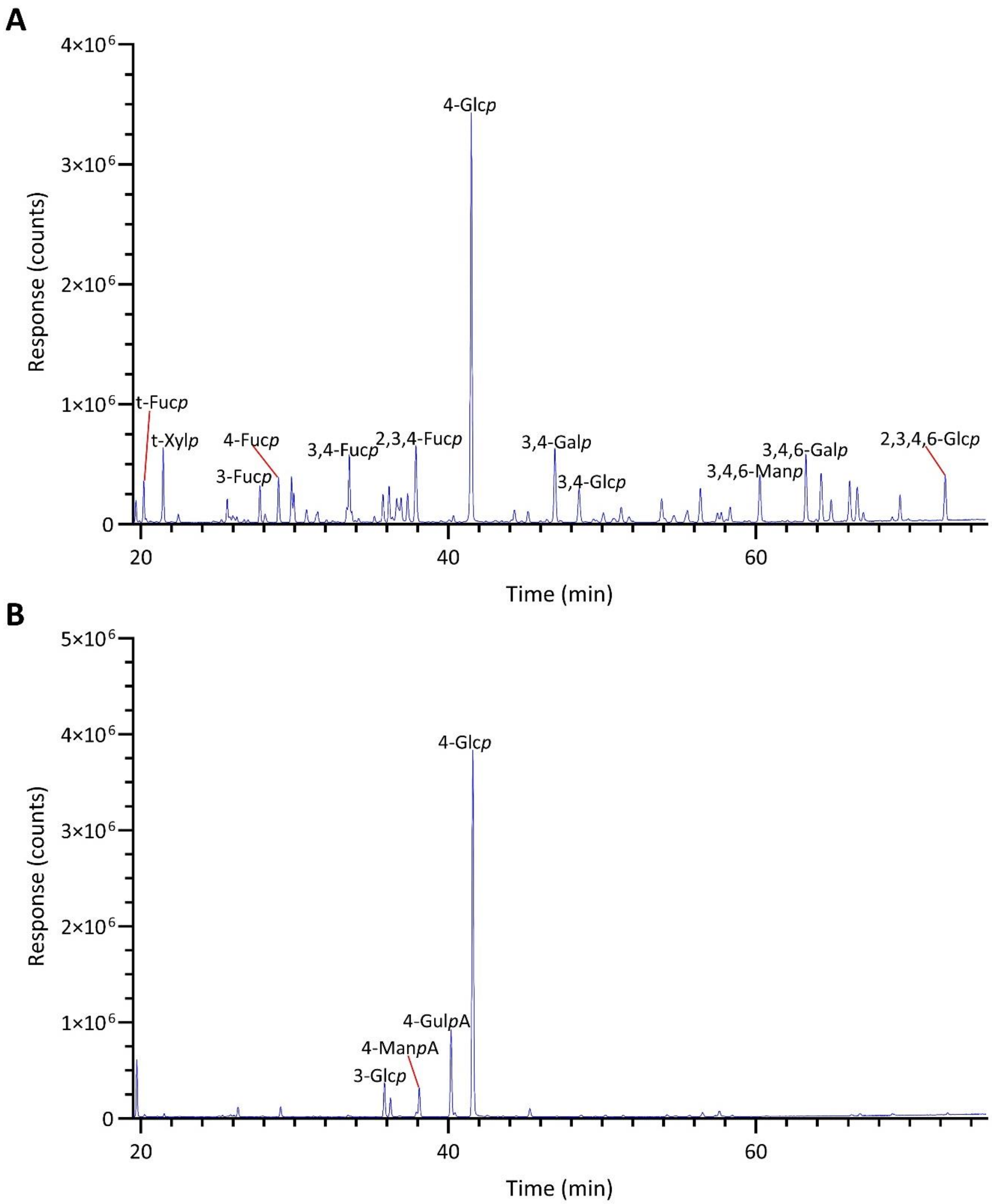

Figure S6. GC-TIC chromatograms of PMAAs from the AIRs of blanched AM harvested in 2022: (**A**) without the pretreatment of weak methanolysis-sodium borodeuteride reduction before methylation, and (**B**) pretreated with weak methanolysis-sodium borodeuteride reduction before methylation.

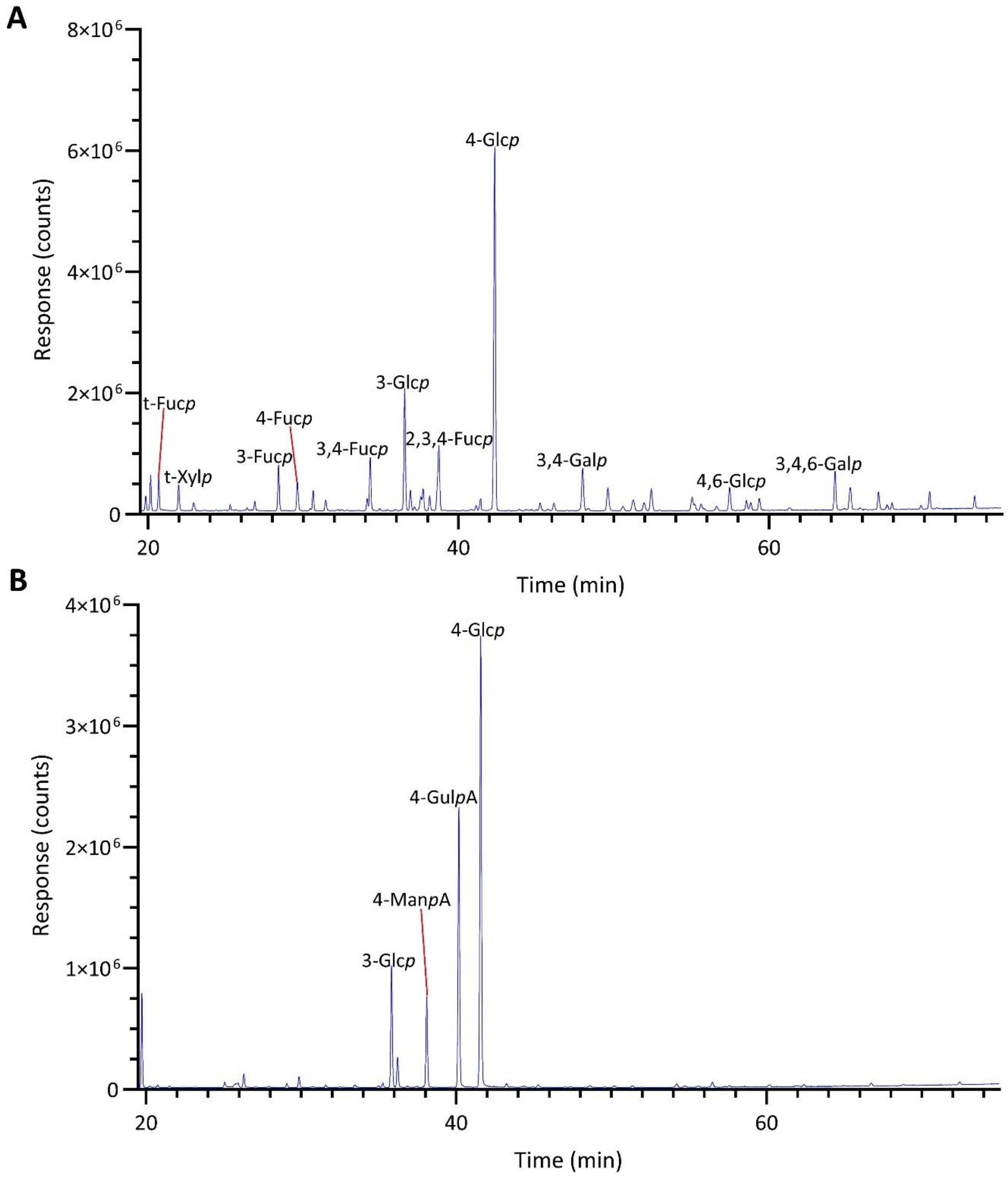

Figure S7. GC-TIC chromatograms of PMAAs from the AIRs of blanched AM harvested in 2021: (**A**) without the pretreatment of weak methanolysis-sodium borodeuteride reduction before methylation, and (**B**) pretreated with weak methanolysis-sodium borodeuteride reduction before methylation.

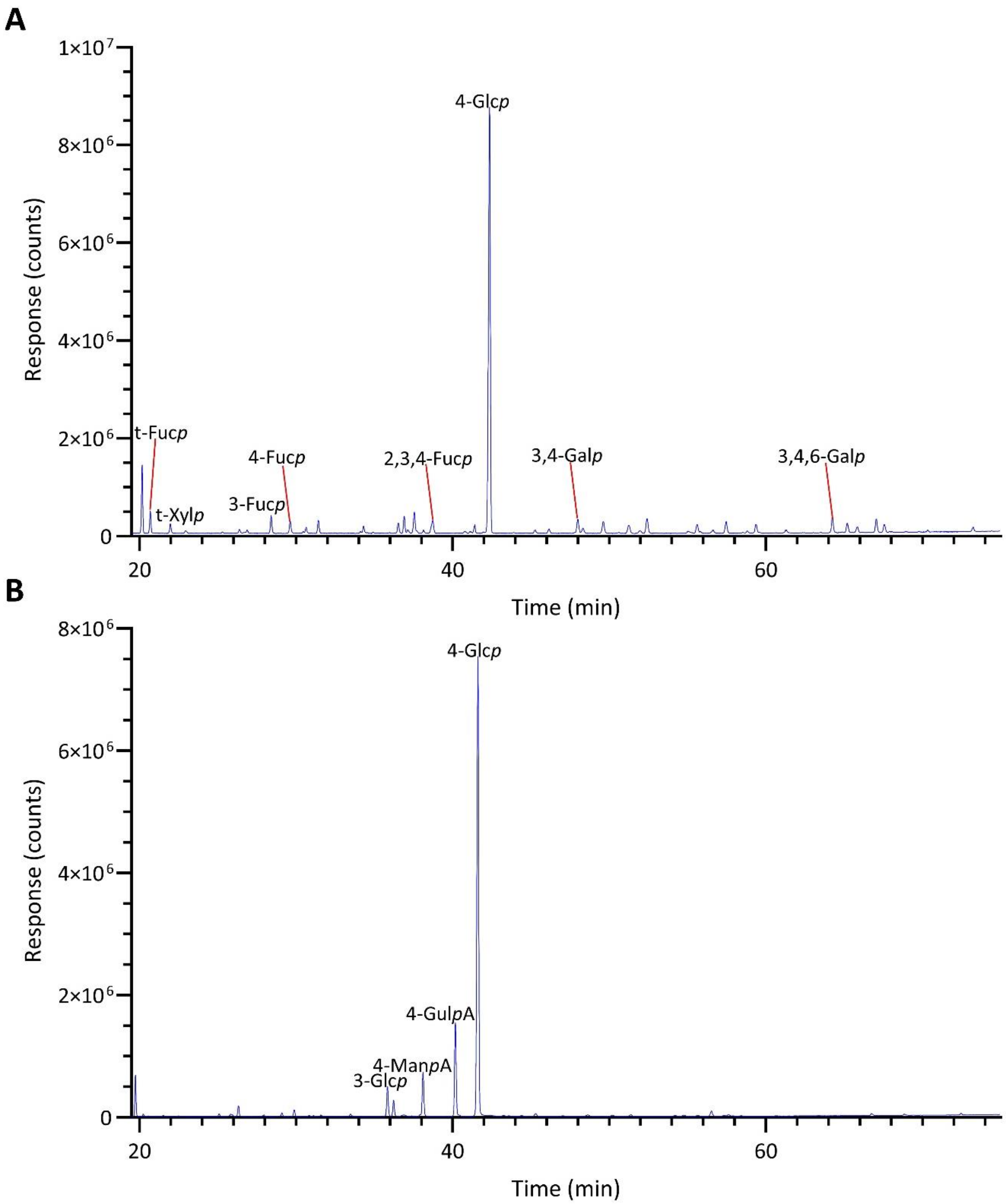

Figure S8. GC-TIC chromatograms of PMAAs from the AIRs of blanched SL harvested in 2021: (**A**) without the pretreatment of weak methanolysis-sodium borodeuteride reduction before methylation, and (**B**) pretreated with weak methanolysis-sodium borodeuteride reduction before methylation.

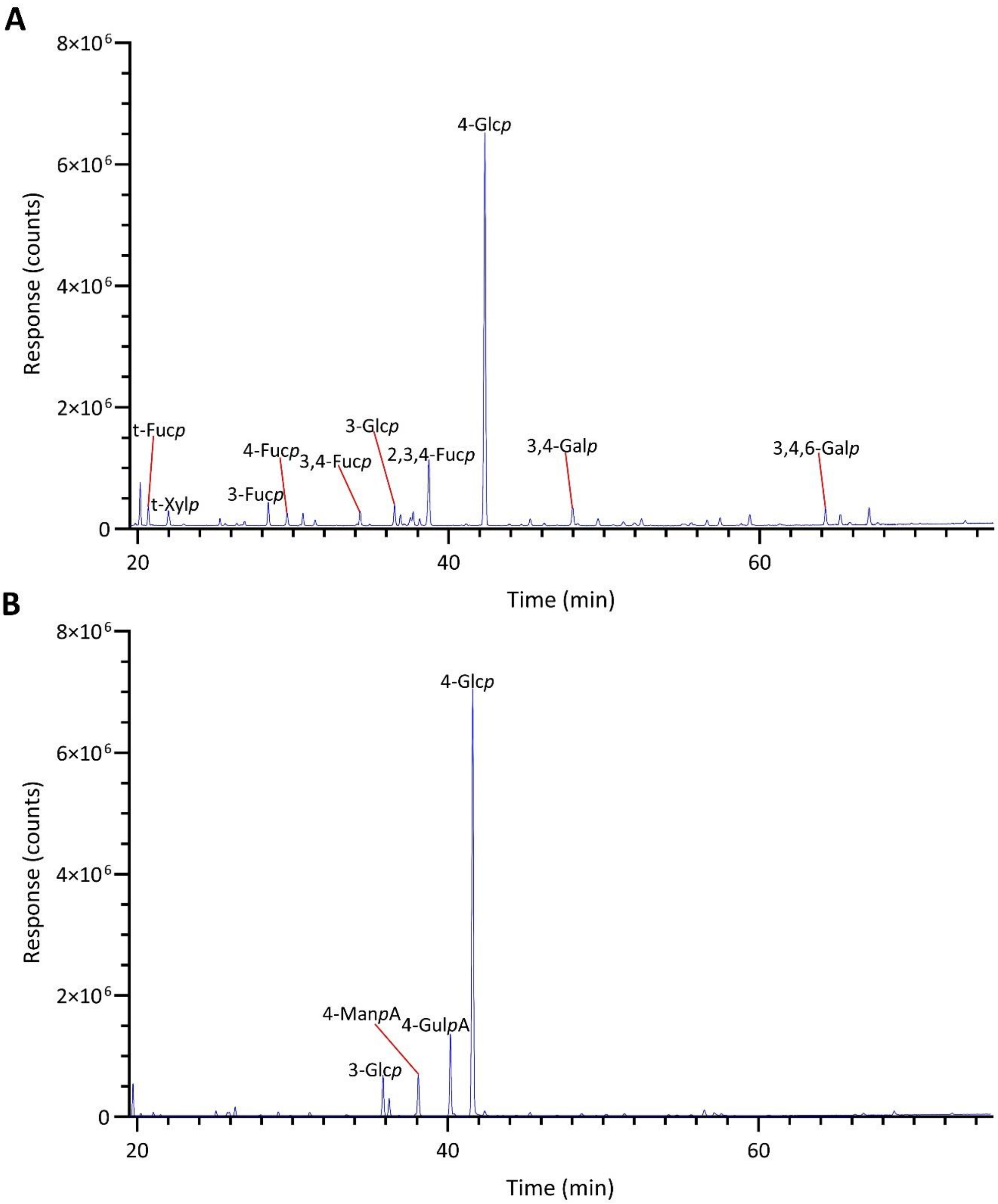

Figure S9. GC-TIC chromatograms of PMAAs from the AIRs of blanched blades of MT: (**A**) without the pretreatment of weak methanolysis-sodium borodeuteride reduction before methylation, and (**B**) pretreated with weak methanolysis-sodium borodeuteride reduction before methylation.

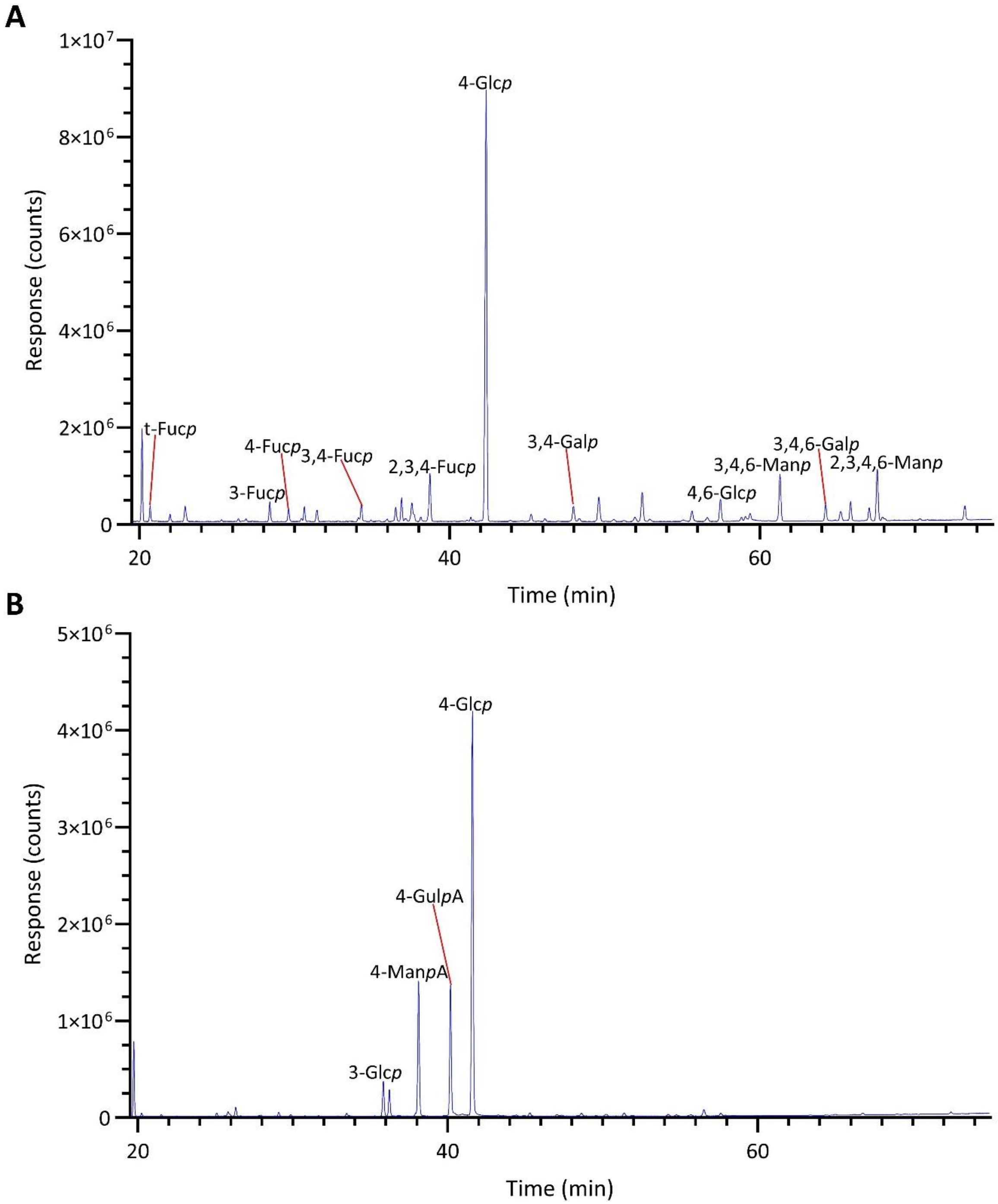

Figure S10. GC-TIC chromatograms of PMAAs from the AIRs of blanched stipes of MT: (**A**) without the pretreatment of weak methanolysis-sodium borodeuteride reduction before methylation, and (**B**) pretreated with weak methanolysis-sodium borodeuteride reduction before methylation.

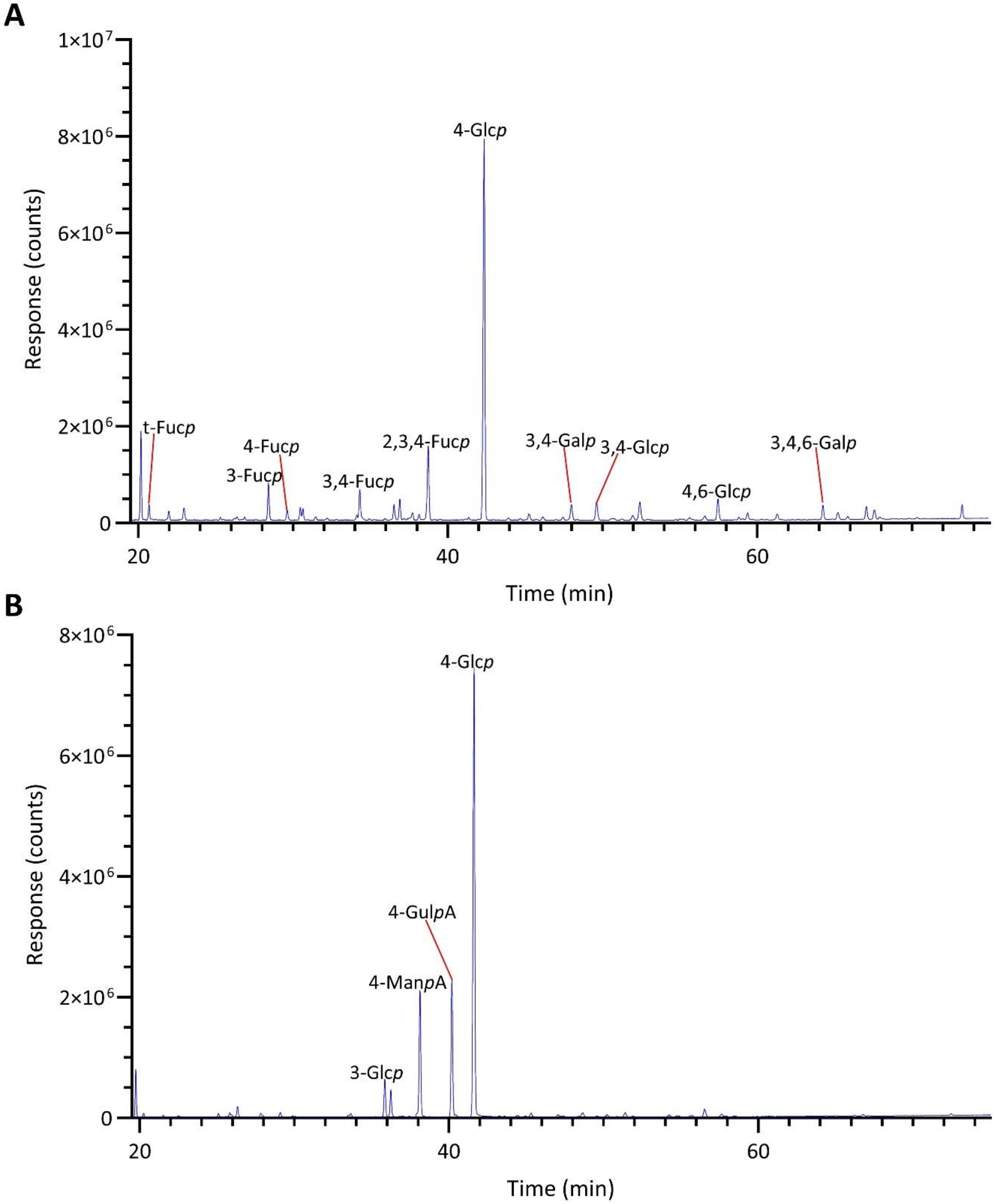

Figure S11. GC-TIC chromatograms of PMAAs from the AIRs of unblanched stipes of MT: (**A**) without the pretreatment of weak methanolysis-sodium borodeuteride reduction before methylation, and (**B**) pretreated with weak methanolysis-sodium borodeuteride reduction before methylation.

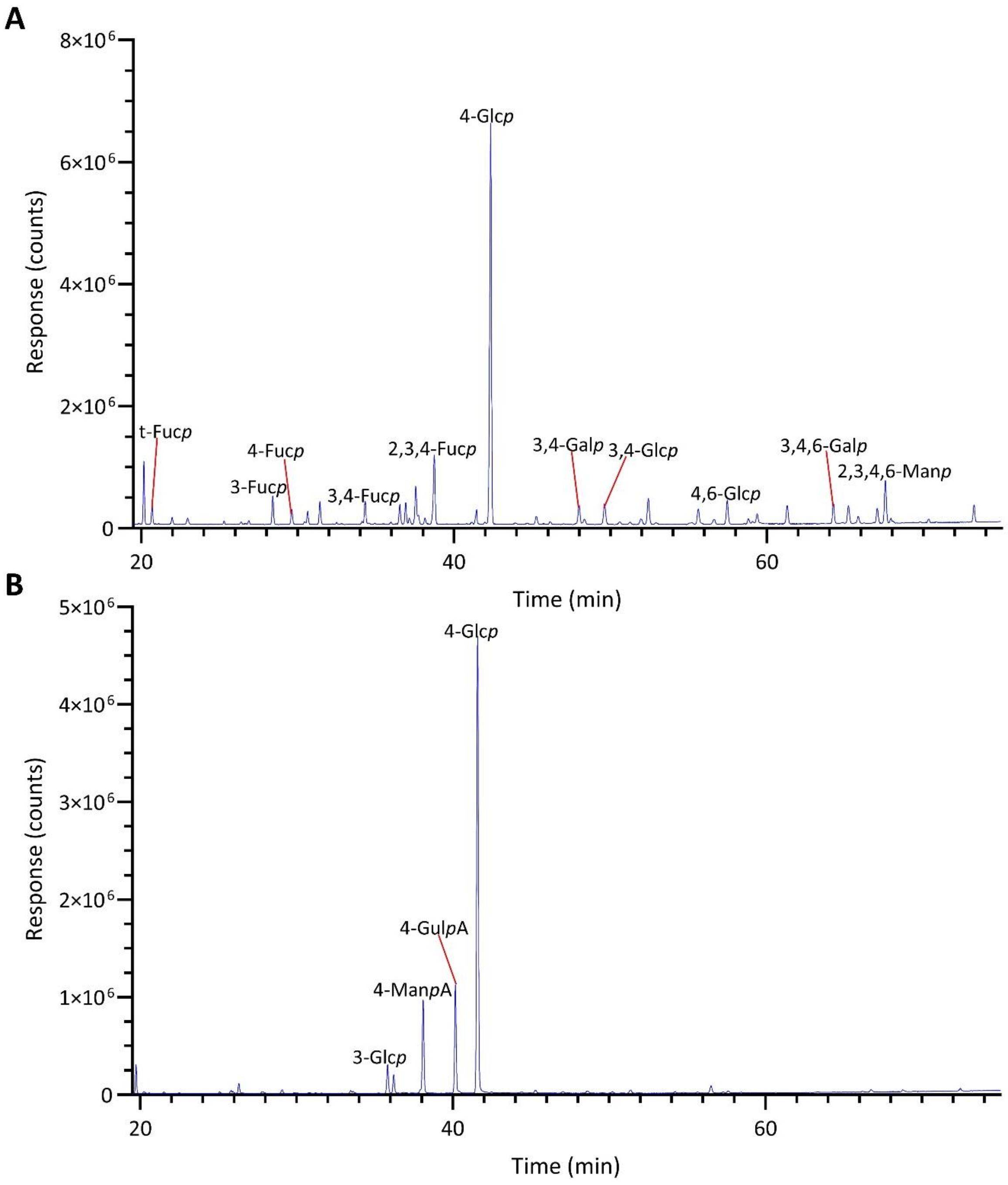

Figure S12. GC-TIC chromatograms of PMAAs from the AIRs of blanched receptacles of MT: (**A**) without the pretreatment of weak methanolysis-sodium borodeuteride reduction before methylation, and (**B**) pretreated with weak methanolysis-sodium borodeuteride reduction before methylation.

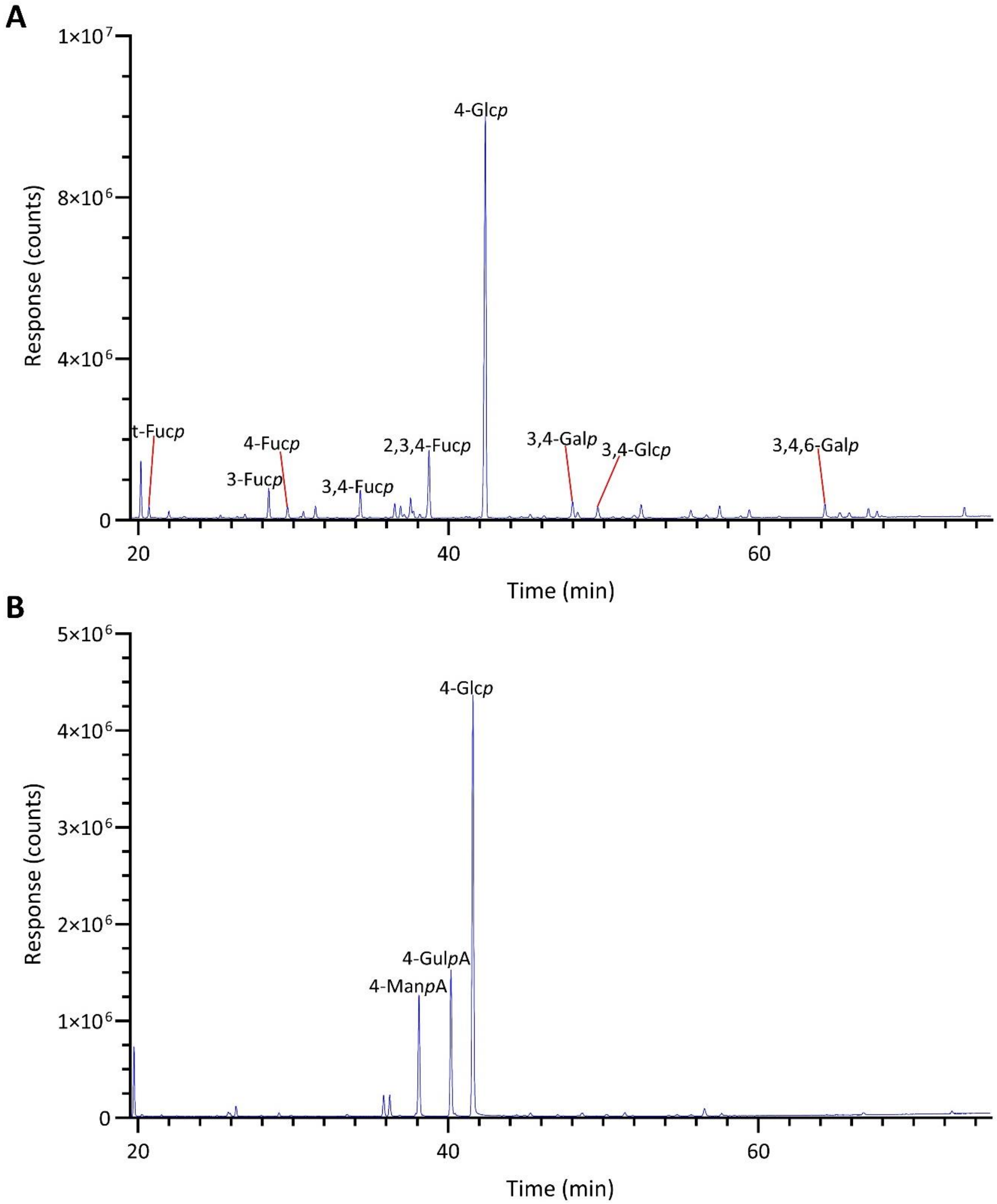

Figure S13. GC-TIC chromatograms of PMAAs from the AIRs of unblanched receptacles of MT: (**A**) without the pretreatment of weak methanolysis-sodium borodeuteride reduction before methylation, and (**B**) pretreated with weak methanolysis-sodium borodeuteride reduction before methylation.

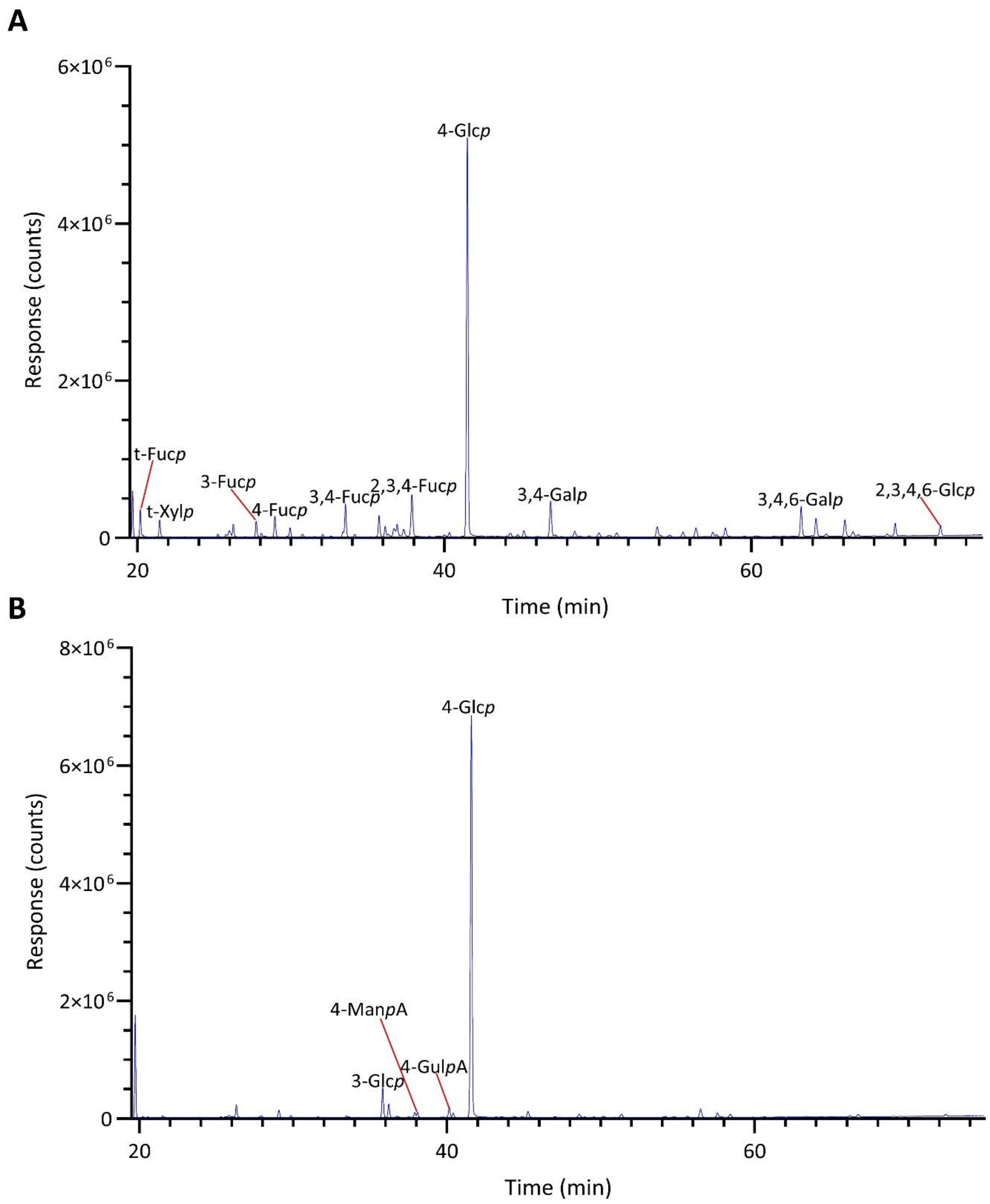

Figure S14. GC-TIC chromatograms of PMAAs from the AIRs of unblanched AM harvested in 2022: (**A**) without the pretreatment of weak methanolysis-sodium borodeuteride reduction before methylation, and (**B**) pretreated with weak methanolysis-sodium borodeuteride reduction before methylation.

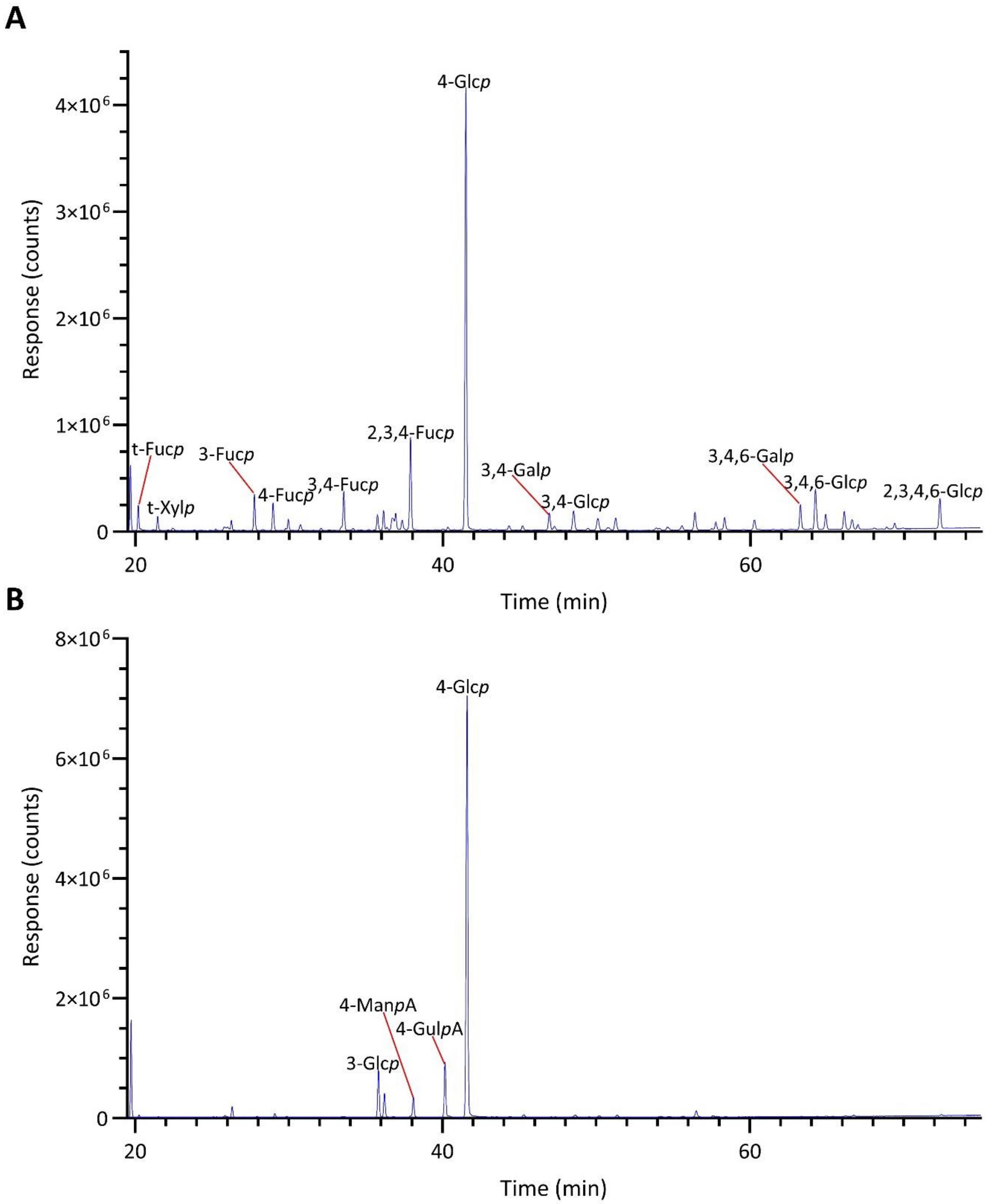

Figure S15. GC-TIC chromatograms of PMAAs from the AIRs of unblanched SL harvested in 2022: (**A**) without the pretreatment of weak methanolysis-sodium borodeuteride reduction before methylation, and (**B**) pretreated with weak methanolysis-sodium borodeuteride reduction before methylation.

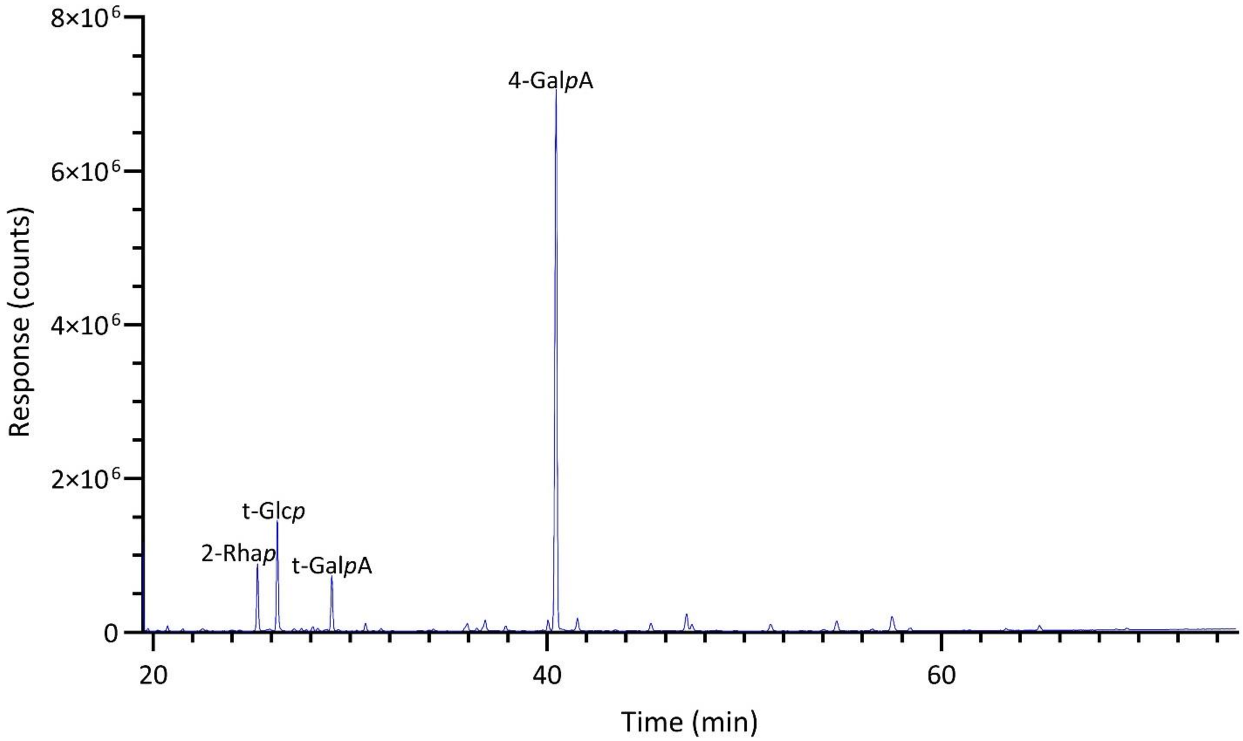

Figure S16. GC-TIC chromatogram of PMAAs derived from commercial polygalacturonic acid sodium salt (Cat. No. P3850, Sigma-Aldrich Co LLC, Massachusetts, USA). The commercial product was subjected to weak methanolysis-sodium borodeuteride reduction followed by methylation-GC-MS analysis.

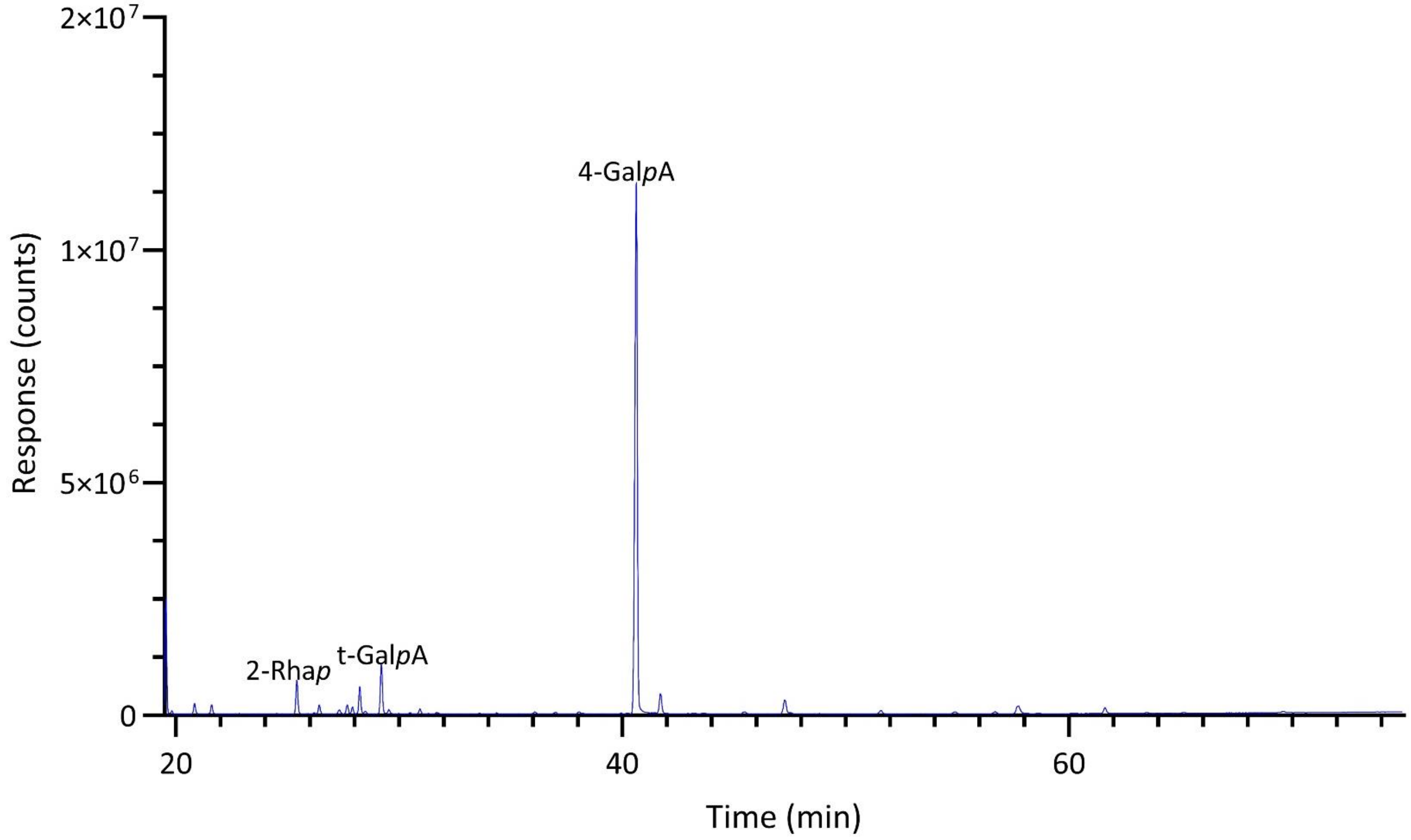

Figure S17. GC-TIC chromatogram of PMAAs derived from commercial apple pectin (Cat. No. P8471, Sigma-Aldrich Co LLC, Massachusetts, USA). The commercial product was subjected to weak methanolysis-sodium borodeuteride reduction followed by methylation-GC-MS analysis.

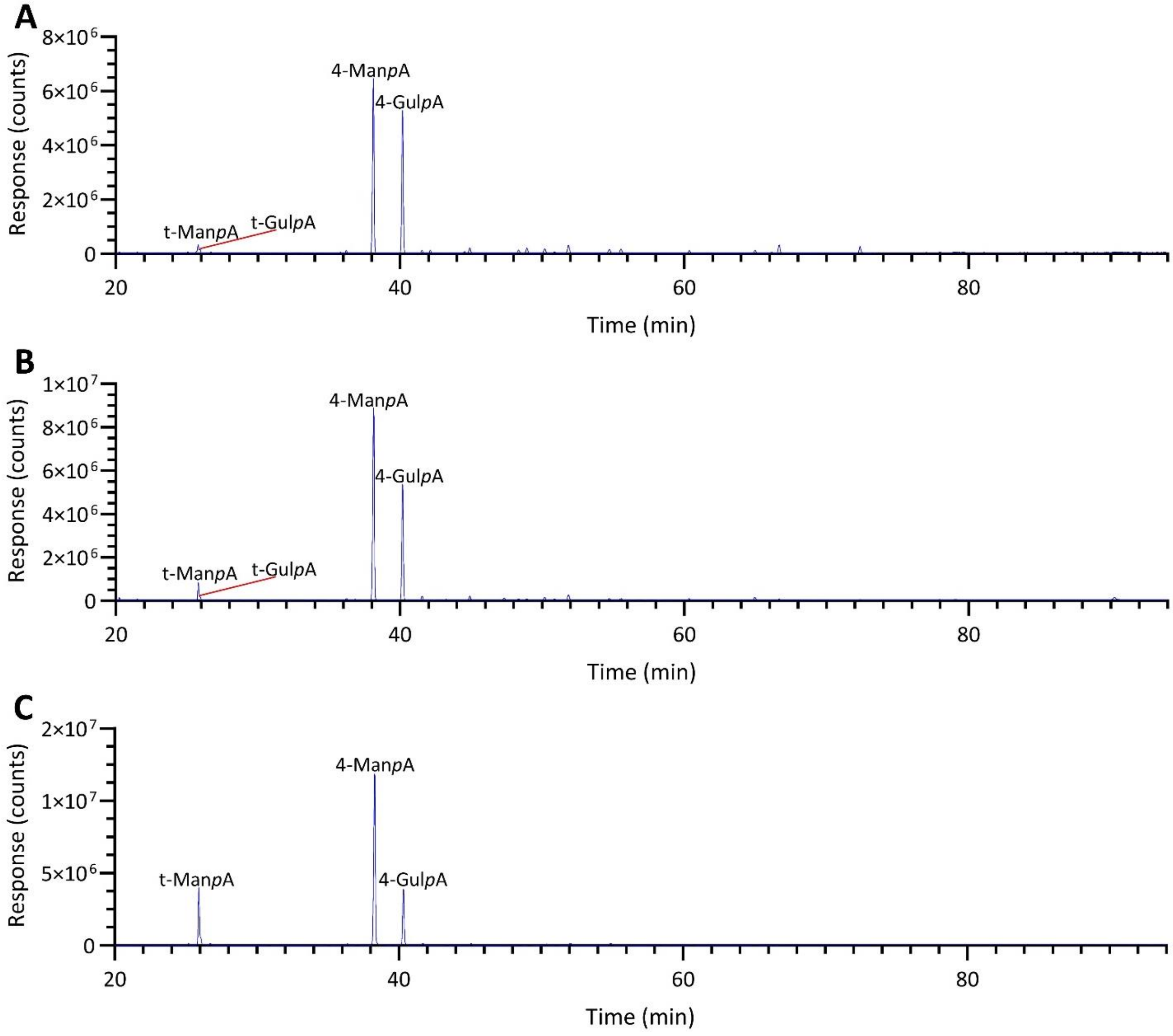

Figure S18. GC-TIC chromatogram of PMAAs derived from commercial alginate standards including (A) alginic acid sodium salt (Cat. No. A2033, Sigma-Aldrich Co LLC, Massachusetts, USA), (B) alginic acid sodium salt (Cat. No. A1112-100G, Sigma-Aldrich Co LLC, Massachusetts, USA), (C) polymannuronic acid sodium salt (Cat. No. YP31737, Biosynth International Inc., Illinois, USA). Each commercial product was subjected to weak methanolysis-sodium borodeuteride reduction followed by methylation-GC-MS analysis. It is apparent that the polymannuronic acid is not pure, as a considerable amount of 4-Gul*p*A was determined.

Table S1. Relative monosaccharide composition (Mol%) of unfractionated polysaccharides of AM, SL, FV, and HE, and the receptacle, blade, and stipe of MT.

Monosaccharides	HE	FV	AM	SL	MT		
					Receptacle	Blade	Stipe
Ara	Trace	Trace	Trace	Trace	Trace	Trace	Trace
Fuc	30.9±0.2	54.0±2.3	12.1±0.3	13.8±2.1	14.8±0.9	19.5±0.1	14.1±2.4
Gal	3.7±0.3	3.4±0.2	8.2±0.2	6.9±2.4	5.4±0.4	7.3±0.6	4.4±0.7
Glc	30.5±2.1	15.5±1.4	47.8±0.9	54.4±2.5	49.4±5.3	54.5±1.2	46.3±0.6
Man	6.3±2.1	4.4±0.1	4.4±0.5	6.1±2.4	4.8±0.5	6.5±0.5	5.2±2.1
Rha	2.1±0.4	1.3±0.2	4.7±0.0	1.0±0.3	0.7±0.0	0.8±0.0	0.7±0.1
Xyl	7.3±0.4	4.9±0.1	2.2±0.4	1.7±0.2	1.4±0.1	2.2±0.2	1.6±0.3
UA	19.2±0.5	16.4±4.0	20.9±0.5	16.0±0.7	23.6±5.9	9.0±1.0	27.7±5.2

Note: Trace means Mol% < 0.5%. AM, SL, and MT were harvested in 2021. FV and HE were harvested in 2020. All samples were unblanched. Two separate experiments were conducted to each sample.

Table S2. Relative polysaccharide composition (Mol%) of unfractionated polysaccharides from the whole plants of AM, SL, FV, and HE, and the receptacle, blade, and stipe of MT.

Linkages	HE	FV	AM	SL	MT		
					Receptacle	Blade	Stipe
AL	17.1±0.5	12.7±3.6	16.8±0.4	11.2±0.2	20.3±5.5	6.7±0.8	24.4±5.1
CE	13.8±0.4	7.3±1.5	23.3±0.4	45.0±3.4	41.9±5.0	46.5±1.1	37.6±2.0
LM	8.9±1.1	5.0±0.8	19.9±0.5	1.5±0.0	1.2±0.2	1.4±0.0	1.4±0.2
NA	29.2±1.2	21.0±1.0	27.8±0.2	28.4±5.7	21.8±0.6	25.9±0.2	22.5±4.9
SF	30.9±0.2	54.0±2.3	12.1±0.3	13.8±2.1	14.8±0.9	19.5±0.1	14.1±2.4

Note: Trace means Mol% < 0.5%. AM, SL, and MT were harvested in 2021. FV and HE were harvested in 2020. All samples were unblanched. Two separate experiments were conducted to each sample.

Table S3. Assignments of linkages for estimation of polysaccharide compositions of brown seaweeds

Polysaccharide	Linkage assignments
AL	Sum of 4-Gul*p*A, 4-Man*p*A, t-Gul*p*A, and t-Man*p*A
CE	All 4-Glc*p*
LM	Sum of 3-Glc*p*, 3,6-Glc*p*, and t-Glc*p* (same amount as 3,6-Glc*p*)
NA	All remaining unassigned, including t-Glc*p* (excluding those assigned to LM)
SF	Sum of all Fuc*p* linkages

Note: CE: cellulose; LM: laminarin; SF: sulfated fucan; AL: alginate; NA: non-assigned linkages

Table S4. Relative glycosidic linkage composition (Mol%) of unfractionated polysaccharides from AM and SL harvested in spring in 2021 and 2022.

Linkages	AM		SL	
	2021	2022	2021	2022
3-Ara*f*	Trace	Trace	Trace	Trace
5-Ara*f*	Trace	Trace	Trace	Trace
t-Fuc*p*	1.7±0.1	3.1±0.5	1.8±0.7	2.3±0.6
2-Fuc*p*	1.0±0.1	1.1±0.1	0.8±0.0	1.0±0.3
3-Fuc*p*	2.8±0.1	1.8±0.2	2.7±0.6	2.9±0.2
4-Fuc*p*	1.0±0.2	2.2±0.2	1.6±0.1	1.7±0.4
2,3-Fuc*p*	1.0±0.1	1.7±0.3	0.8±0.6	1.3±0.3
2,4-Fuc*p*	0.6±0.1	1.1±0.2	Trace	0.9±0.2
3,4-Fuc*p*	2.3±0.2	3.6±0.3	2.6±1.9	2.2±0.8
2,3,4-Fuc*p*	1.7±0.2	3.2±0.3	3.1±0.1	5.5±0.6
t-Gal*p*	Trace	Trace	Trace	Trace
2-Gal*p*	1.0±0.0	Trace	0.7±0.1	Trace
4-Gal*p*	Trace	Trace	Trace	Trace
6-Gal*p*	Trace	0.9±0.1	Trace	Trace
3,4-Gal*p*	3.3±0.4	5.0±0.8	1.9±0.2	1.6±0.0
3,6-Gal*p*	0.6±0.0	1.1±0.1	1.2±0.5	1.0±0.1
4,6-Gal*p*	Trace	0.6±0.1	Trace	Trace
2,3,6-Gal*p*	Trace	Trace	Trace	Trace
2,4,6-Gal*p*	Trace	Trace	Trace	Trace
3,4,6-Gal*p*	1.3±0.1	3.3±0.4	1.8±0.7	1.7±0.2
2,3,4,6-Gal*p*	Trace	1.0±0.1	Trace	Trace
2,4-Glc*p*+2,4-Gal*p*	0.8±0.0	0.6±0.1	1.8±0.7	1.2±0.1
t-Glc*p*	1.4±0.1	1.2±0.3	Trace	0.7±0.0
3-Glc*p*	18.7±0.2	1.6±0.1	1.2±0.1	0.9±0.0
4-Glc*p*	23.3±0.3	40.5±4.6	45.0±3.4	40.5±6.1
6-Glc*p*	Trace	Trace	Trace	Trace
2,3-Glc*p*	Trace	Trace	Trace	Trace
3,4-Glc*p*	0.7±0.1	0.8±0.2	1.5±0.1	1.5±0.3
3,6-Glc*p*	0.6±0.1	Trace	Trace	Trace
4,6-Glc*p*	Trace	1.1±0.1	1.4±0.1	1.4±0.1
2,3,6-Glc*p*	Trace	Trace	Trace	Trace
2,4,6-Glc*p*	Trace	Trace	Trace	Trace
3,4,6-Glc*p*	0.7±0.3	2.0±0.4	1.2±0.6	2.5±1.1
2,3,4,6-Glc*p*	Trace	0.7±0.2	1.0±0.9	1.4±0.9
t-Man*p*	Trace	Trace	Trace	Trace
2-Man*p*	1.0±0.1	0.7±0.2	1.2±0.1	1.3±0.0
3-Man*p*	Trace	Trace	Trace	Trace

4-Man*p*	Trace	0.7±0.1	Trace	Trace
2,3-Man*p*	Trace	Trace	Trace	Trace
2,4-Man*p*	0.8±0.0	0.7±0.1	0.6±0.7	1.2±0.0
2,6-Man*p*	Trace	0.7±0.1	Trace	Trace
3,4-Man*p*	Trace	Trace	Trace	Trace
3,6-Man*p*	Trace	Trace	Trace	Trace
4,6-Man*p*	Trace	Trace	Trace	Trace
2,3,6-Man*p*	0.6±0.1	0.8±0.1	1.0±0.6	0.7±0.1
2,4,6-Man*p*	Trace	1.0±0.2	Trace	0.6±0.1
3,4,6-Man*p*	Trace	Trace	Trace	Trace
2,3,4,6-Man*p*	Trace	Trace	Trace	Trace
t-Rha*p*	Trace	Trace	Trace	Trace
2-Rha*p*	Trace	Trace	Trace	Trace
3-Rha*p*	Trace	0.8±0.0	Trace	Trace
4-Rha*p*	Trace	Trace	Trace	Trace
2,3-Rha*p*	Trace	Trace	Trace	Trace
2,4-Rha*p*	4.3±0.1	Trace	Trace	Trace
3,4-Rha*p*	Trace	Trace	Trace	Trace
2,3,4-Rha*p*	Trace	Trace	Trace	Trace
t-Xyl*p*	1.4±0.2	2.2±0.3	1.0±0.1	1.3±0.2
2-Xyl*p*	Trace	Trace	Trace	Trace
3-Xyl*p*	Trace	Trace	Trace	Trace
4-Xyl*p*	Trace	Trace	Trace	Trace
2,4-Xyl*p*	Trace	Trace	Trace	Trace
3,4-Xyl*p*	Trace	Trace	Trace	Trace
2,3,4-Xyl*p*	Trace	Trace	Trace	Trace
t-Gal*p*A	Trace	Trace	Trace	Trace
2,4-Glc*p*A+2,4-Gal*p*A	Trace	Trace	Trace	Trace
t-Glc*p*A	Trace	Trace	Trace	Trace
3-Glc*p*A	1.8±0.3	0.8±0.2	2.1±0.1	3.1±0.3
4-Glc*p*A	2.0±0.5	1.8±0.4	2.5±0.3	2.6±0.2
t-Gul*p*A	Trace	Trace	Trace	Trace
4-Gul*p*A	12.4±0.7	2.5±0.5	8.1±0.3	6.4±1.1
t-Man*p*A	Trace	Trace	Trace	Trace
4-Man*p*A	4.1±0.2	Trace	2.8±0.1	2.1±0.4

Note: Trace means Mol% < 0.5%. Samples were unblanched. For each species, three separate experiments were conducted on those harvested in 2022, and two were conducted on those harvested in 2021.

Table S5. Relative monosaccharide composition (Mol%) of unfractionated polysaccharides from AM and SL harvested in spring in 2021 and 2022.

Monosaccharides	AM		SL	
	2021	2022	2021	2022
Ara	Trace	Trace	Trace	Trace
Fuc	12.1±0.3	17.8±1.7	13.8±2.1	17.9±1.1
Gal	8.2±0.2	13.0±1.6	6.9±2.4	5.7±0.4
Glc	47.8±0.9	49.4±4.1	54.4±2.5	51.2±3.5
Man	4.4±0.5	7.1±1.0	6.1±2.4	6.8±1.0
Rha	4.7±0.0	3.0±0.1	1.0±0.3	1.5±0.1
Xyl	2.2±0.4	3.5±0.3	1.7±0.2	2.1±0.1
UA	20.9±0.5	6.2±0.4	16.0±0.7	14.8±1.4

Note: Trace means Mol% < 0.5%. Samples were unblanched. For each species, three separate experiments were conducted on those harvested in 2022, and two were conducted on those harvested in 2021.

Table S6. Relative polysaccharide composition (Mol%) of unfractionated polysaccharides from AM and SL harvested in spring in 2021 and 2022.

Linkages	AM		SL	
	2021	2022	2021	2022
AL	16.8±0.4	3.2±0.7	11.2±0.2	8.7±1.6
CE	23.3±0.4	40.5±4.6	45.0±3.4	40.5±6.1
LM	19.9±0.5	2.0±0.1	1.5±0.0	1.3±0.1
NA	27.8±0.2	36.5±2.4	28.4±5.7	31.6±3.8
SF	12.1±0.3	17.8±1.7	13.8±2.1	17.9±1.1

Note: Samples were unblanched. For each species, three separate experiments were conducted on those harvested in 2022, and two were conducted on those harvested in 2021.

Table S7. Relative glycosidic linkage composition (Mol%) of unfractionated polysaccharides of AM and SL, and the receptacle, blade, and stipe of MT , with and without blanching treatments.

Linkage	AM		SL		MT					
					Receptacle		Blade		Stipe	
	U	B	U	B	U	B	U	B	U	B
3-Ara*f*	Trace	Trace	Trace	Trace	Trace	Trace	Trace	Trace	Trace	Trace
5-Ara*f*	Trace	Trace	Trace	Trace	Trace	Trace	Trace	Trace	Trace	Trace
t-Fuc*p*	1.7±0.1	2±0.1	1.8±0.7	2.0±0.3	1.2±0.1	1.4±0.1	1.6±0.2	2.1±0.3	1.2±0.2	1.3±0.1
2-Fuc*p*	1±0.1	1.1±0.2	0.8±0.0	0.6±0.1	0.7±0.0	1.0±0.0	1.1±0.1	1.5±0.4	0.8±0.2	1.0±0.4
3-Fuc*p*	2.8±0.1	3.1±0.5	2.7±0.6	1.7±0.2	2.7±0.2	2.5±0.5	3.0±0.5	2.7±0.4	2.6±0.7	1.7±0.1
4-Fuc*p*	1±0.2	1.5±0.3	1.6±0.1	1.1±0.2	1.0±0.1	1.2±0.1	1.4±0.2	1.8±0.5	0.9±0.0	1.0±0.3
2,3-Fuc*p*	1±0.1	1.3±0.1	0.8±0.6	Trace	0.7±0.0	0.8±0.2	1.5±0.0	1.7±0.4	0.7±0.1	Trace
2,4-Fuc*p*	0.6±0.1	0.8±0.2	Trace	Trace	Trace	0.6±0.1	0.7±0.0	1.0±0.3	Trace	Trace
3,4-Fuc*p*	2.3±0.2	2.9±0.3	2.6±1.9	0.7±0.1	2.2±0.6	1.8±0.2	2.5±0.6	2.1±0.8	2.5±0.2	1.7±0.2
2,3,4-Fuc*p*	1.7±0.2	2.7±0.9	3.1±0.1	0.9±0.1	5.8±0.1	5.4±1.3	7.7±0.1	7.4±2.2	4.9±0.8	2.9±1.2
t-Gal*p*	Trace	Trace	Trace	Trace	Trace	Trace	Trace	Trace	Trace	Trace
2-Gal*p*	1±0	1.1±0.1	0.7±0.1	Trace	0.6±0.0	0.6±0.1	0.6±0.0	0.7±0.1	Trace	Trace
4-Gal*p*	Trace	Trace	Trace	Trace	Trace	Trace	Trace	Trace	Trace	Trace
6-Gal*p*	Trace	Trace	Trace	Trace	Trace	Trace	Trace	Trace	Trace	Trace
3,4-Gal*p*	3.3±0.4	4±1.4	1.9±0.2	1.7±0.2	1.9±0.1	2.0±0.4	2.6±0.2	2.5±0.3	1.5±0.2	1.5±0.2
3,6-Gal*p*	0.6±0	0.8±0	1.2±0.5	1.0±0.1	0.9±0	1.0±0.1	1.4±0.2	1.4±0.3	0.6±0.1	0.7±0.2
4,6-Gal*p*	Trace	0.6±0.1	Trace	Trace	Trace	Trace	Trace	Trace	Trace	Trace
2,3,6-Gal*p*	Trace	Trace	Trace	Trace	Trace	Trace	Trace	Trace	Trace	Trace
2,4,6-Gal*p*	Trace	Trace	Trace	Trace	Trace	Trace	Trace	Trace	Trace	Trace
3,4,6-Gal*p*	1.3±0.1	2±0.5	1.8±0.7	1.6±0.1	1.3±0.2	1.5±0.0	1.8±0.2	2.3±0.8	1.1±0.1	1.2±0.4
2,3,4,6-Gal*p*	Trace	0.8±0.2	Trace	Trace	Trace	Trace	Trace	Trace	Trace	Trace
2,4-Glc*p*+2,4-Gal*p*	0.8±0	1.2±0.5	1.8±0.7	1.7±0.3	1.7±0.2	1.6±1.1	1.5±0.1	1.2±0.4	1.8±0.3	2.4±1.4
t-Glc*p*	1.4±0.1	0.6±0	Trace	Trace	Trace	Trace	Trace	Trace	Trace	Trace
3-Glc*p*	18.7±0.2	6.1±0.5	1.2±0.1	1.0±0.2	0.9±0.2	1.2±0.2	1.1±0.0	1.7±0.1	1.2±0.2	1.4±0.5
4-Glc*p*	23.3±0.4	25.4±2.3	45.0±3.4	51.9±3.2	41.9±5.0	37.7±7.8	46.5±1.1	34.8±11.9	37.6±2	43.3±2.3

6-Glc*p*	Trace	Trace	Trace	Trace	Trace	Trace	Trace	Trace	Trace	Trace
2,3-Glc*p*	Trace	Trace	Trace	Trace	Trace	Trace	Trace	Trace	Trace	Trace
3,4-Glc*p*	0.7±0.1	1.3±0.6	1.5±0.1	1.5±0.1	1.4±0.0	1.5±0.6	1.3±0.1	1.2±0.4	1.7±0.4	2.0±1.2
3,6-Glc*p*	0.6±0.1	Trace	Trace	Trace	Trace	Trace	Trace	Trace	Trace	Trace
4,6-Glc*p*	Trace	1.1±0.6	1.4±0.1	1.3±0.0	1.3±0.0	1.4±0.7	1.4±0.0	1.1±0.3	1.6±0.6	1.7±0.7
2,3,6-Glc*p*	Trace	Trace	Trace	Trace	Trace	Trace	Trace	Trace	Trace	Trace
2,4,6-Glc*p*	Trace	Trace	Trace	Trace	Trace	Trace	Trace	Trace	Trace	Trace
3,4,6-Glc*p*	0.7±0.3	1.1±0.7	1.2±0.6	1.0±0.1	0.6±0.0	0.9±0.8	1.5±0.1	1.6±0.6	0.7±0.1	0.7±0.4
2,3,4,6-Glc*p*	Trace	0.6±0.1	1.0±0.9	Trace	0.6±0.3	0.7±0.6	Trace	0.6±0.5	0.8±0.3	0.8±0.7
t-Man*p*	Trace	Trace	Trace	Trace	Trace	Trace	Trace	Trace	Trace	Trace
2-Man*p*	1±0.1	1.1±0.1	1.2±0.1	1.4±0.2	1.0±0.0	1.1±0.5	0.9±0.1	1.1±0.2	1.3±0.5	1.5±0.5
3-Man*p*	Trace	Trace	Trace	Trace	Trace	Trace	Trace	Trace	Trace	Trace
4-Man*p*	Trace	Trace	Trace	Trace	Trace	Trace	0.7±0.0	0.6±0.1	Trace	Trace
2,3-Man*p*	Trace	Trace	Trace	Trace	Trace	Trace	0.7±0.1	0.8±0.2	Trace	0.6±0.2
2,4-Man*p*	0.8±0	1±0	0.6±0.7	1.1±0.2	Trace	Trace	Trace	0.6±0.0	Trace	Trace
2,6-Man*p*	Trace	Trace	Trace	Trace	Trace	Trace	0.7±0.1	0.8±0.2	Trace	Trace
3,4-Man*p*	Trace	Trace	Trace	Trace	Trace	Trace	Trace	Trace	Trace	Trace
3,6-Man*p*	Trace	Trace	Trace	Trace	Trace	Trace	Trace	Trace	Trace	Trace
4,6-Man*p*	Trace	Trace	Trace	Trace	Trace	Trace	Trace	Trace	Trace	Trace
2,3,6-Man*p*	0.6±0.1	0.8±0	1.0±0.6	1.0±0.1	0.6±0.0	0.7±0.0	0.9±0.0	0.9±0.1	0.7±0.2	0.7±0.2
2,4,6-Man*p*	Trace	Trace	Trace	Trace	Trace	Trace	0.9±0.1	1.0±0.1	Trace	Trace
3,4,6-Man*p*	Trace	Trace	Trace	Trace	Trace	0.9±1.0	Trace	Trace	Trace	2.3±3.0
2,3,4,6-Man*p*	Trace	Trace	Trace	Trace	0.8±0.4	1.6±1.9	Trace	Trace	0.6±0.4	2.3±2.8
t-Rha*p*	Trace	Trace	Trace	Trace	Trace	Trace	Trace	Trace	Trace	Trace
2-Rha*p*	Trace	Trace	Trace	Trace	Trace	Trace	Trace	Trace	Trace	Trace
3-Rha*p*	Trace	Trace	Trace	Trace	Trace	Trace	Trace	Trace	Trace	Trace
4-Rha*p*	Trace	Trace	Trace	Trace	Trace	Trace	Trace	Trace	Trace	Trace
2,3-Rha*p*	Trace	Trace	Trace	Trace	Trace	Trace	Trace	Trace	Trace	Trace
2,4-Rha*p*	4.3±0.1	1.5±0.2	Trace	Trace	Trace	Trace	Trace	0.6±0.0	Trace	Trace
3,4-Rha*p*	Trace	Trace	Trace	Trace	Trace	Trace	Trace	Trace	Trace	Trace

2,3,4-Rha*p*	Trace	Trace	Trace	Trace	Trace	Trace	Trace	Trace	Trace	Trace
t-Xyl*p*	1.4±0.2	1.7±0.1	1.0±0.1	1.0±0.2	0.8±0.1	0.8±0.1	1.5±0.2	2.4±0.3	0.9±0.1	0.7±0.1
2-Xyl*p*	Trace	Trace	Trace	Trace	Trace	Trace	Trace	Trace	Trace	Trace
3-Xyl*p*	Trace	Trace	Trace	Trace	Trace	Trace	Trace	Trace	Trace	Trace
4-Xyl*p*	Trace	Trace	Trace	Trace	Trace	Trace	Trace	Trace	Trace	Trace
2,4-Xyl*p*	Trace	Trace	Trace	Trace	Trace	Trace	Trace	Trace	Trace	Trace
3,4-Xyl*p*	Trace	Trace	Trace	Trace	Trace	Trace	Trace	Trace	Trace	Trace
2,3,4-Xyl*p*	Trace	Trace	Trace	Trace	Trace	Trace	Trace	Trace	Trace	Trace
t-Gal*p*A	Trace	Trace	Trace	Trace	Trace	Trace	Trace	Trace	Trace	Trace
2,4-Glc*p*A+2,4-Gal*p*A	Trace	Trace	Trace	Trace	Trace	Trace	Trace	Trace	Trace	Trace
t-Glc*p*A	Trace	Trace	Trace	Trace	Trace	Trace	Trace	Trace	Trace	Trace
3-Glc*p*A	1.8±0.3	1.6±0.1	2.1±0.1	1.2±0.3	0.8±0.1	0.8±0.0	0.6±0.2	1.6±0.0	0.8±0.0	0.7±0.5
4-Glc*p*A	2±0.5	2.2±0.5	2.5±0.3	2.2±0.1	2.3±0.2	2.2±0.4	1.6±0.1	2.2±0.5	2.4±0.1	2.2±1.0
t-Gul*p*A	Trace	Trace	Trace	Trace	Trace	Trace	Trace	Trace	Trace	Trace
4-Gul*p*A	12.4±0.7	15.2±2.5	8.1±0.3	10.5±1.6	10.8±2.9	10.3±0.6	5.1±0.7	7.4±0.5	12.1±2.2	8.9±6.5
t-Man*p*A	Trace	Trace	Trace	Trace	Trace	Trace	Trace	Trace	Trace	Trace
4-Man*p*A	4.1±0.2	5.6±0.5	2.8±0.1	3.7±0.9	8.9±2.6	10.4±2.5	1.4±0.2	4.1±0.5	11.9±2.8	7.7±8.5

Note: Trace means Mol% < 0.5%. AM, SL, and MT were harvested in 2021. Two separate experiments were conducted on each sample of *Alaria marginata, Saccharina latissima* and *Macrocystis tenuifolia* .

Table S8. Relative monosaccharide composition (Mol%) of unfractionated polysaccharides from the whole seaweed of AM and SL, and the receptacle, blade, and stipe of MT, with and without blanching treatments.

Monosaccharides	AM		SL		MT					
					Receptacle		Blade		Stipe	
	U	B	U	B	U	B	U	B	U	B
Ara	Trace	Trace	Trace	Trace	Trace	Trace	Trace	Trace	Trace	Trace
Fuc	12.1±0.3	15.3±1.4	13.8±2.1	7.5±1.0	14.8±0.9	14.6±2.1	19.5±0.1	20.3±5.4	14.1±2.4	10.6±2.1
Gal	8.2±0.2	10.5±0.6	6.9±2.4	5.8±0.5	5.4±0.4	6.1±0.5	7.3±0.6	8.1±1.8	4.4±0.7	4.5±1.1
Glc	47.7±0.9	38.5±0	54.4±2.5	59.8±2.7	49.4±5.3	46.2±4.0	54.5±1.2	43.7±9.0	46.3±0.6	53.4±6.0
Man	4.4±0.5	5.5±0.2	6.1±2.4	6.4±1.4	4.8±0.5	6.8±3.9	6.5±0.5	7.5±1.5	5.2±2.1	9.1±7.2
Rha	4.6±0	1.9±0.1	1.0±0.3	0.7±0.0	0.7±0.0	0.7±0.0	0.8±0.0	1.2±0.2	0.7±0.1	0.8±0.0
Xyl	2.2±0.4	2.8±0.5	1.7±0.2	1.5±0.2	1.4±0.1	1.4±0.0	2.2±0.2	3.3±0.8	1.6±0.3	1.4±0.4
UA	20.9±0.5	25.5±1.4	16.0±0.7	18.1±0.5	23.6±5.9	24.0±2.7	9.0±1.0	15.9±0.8	27.7±5.2	20.1±16.8

Note: Trace means Mol% < 0.5%. AM, SL, and MT were harvested in 2021. Two separate experiments were conducted on each sample of AM, SL, and MT.

Table S9. Relative polysaccharide composition (Mol%) of unfractionated polysaccharides from the whole seaweed of AM and SL, and the receptacle, blade, and stipe of MT, with and without blanching treatments.

Linkages	AM		SL		MT					
					Receptacle		Blade		Stipe	
	U	B	U	B	U	B	U	B	U	B
AL	16.9±0.3	21.4±2	11.2±0.2	14.5±0.7	20.3±5.5	21.0±3.1	6.7±0.8	11.9±0.2	24.4±5.1	17.0±15.3
CE	23.3±0.3	25.4±2.3	45.0±3.4	51.9±3.2	41.9±5.0	37.7±7.8	46.5±1.1	34.8±11.9	37.6±2.0	43.3±2.3
LM	19.3±0.3	6.4±0.4	1.5±0.0	1.2±0.2	1.2±0.2	1.5±0.2	1.4±0.0	2.1±0.3	1.4±0.2	1.7±0.6
NA	28.4±0	31.5±3.4	28.4±5.7	24.9±3.1	21.8±0.6	25.1±6.9	25.9±0.2	30.8±6.4	22.5±4.9	27.5±11.5
SF	12.1±0.3	15.3±1.4	13.8±2.1	7.5±1.0	14.8±0.9	14.6±2.1	19.5±0.1	20.3±5.4	14.1±2.4	10.6±2.1

Note: Trace means Mol% < 0.5%. AM, SL, and MT were harvested in 2021. Two separate experiments were conducted on each sample of AM, SL, and MT.

Table S10. Abbreviations of detected PMAAs from the glycosidic linkage analysis of brown seaweed polysaccharides.

Abbreviation	Linkage
3-Ara*f*	3-linked arabinose furanose
5-Ara*f*	5-linked arabinose furanose
t-Fuc*p*	Terminal fucose pyranose
2-Fuc*p*	2-linked fucose pyranose
3-Fuc*p*	3-linked fucose pyranose
4-Fuc*p*	4-linked fucose pyranose
2,3-Fuc*p*	2,3-linked fucose pyranose
2,4-Fuc*p*	2,4-linked fucose pyranose
3,4-Fuc*p*	3,4-linked fucose pyranose
2,3,4-Fuc*p*	2,3,4-linked fucose pyranose
t-Gal*p*	Terminal galactose pyranose
2-Gal*p*	2-linked galactose pyranose
4-Gal*p*	4-linked galactose pyranose
6-Gal*p*	6-linked galactose pyranose
3,4-Gal*p*	3,4-linked galactose pyranose
3,6-Gal*p*	3,6-linked galactose pyranose
4,6-Gal*p*	4,6-linked galactose pyranose
2,3,6-Gal*p*	2,3,6-linked galactose pyranose
2,4,6-Gal*p*	2,4,6-linked galactose pyranose
3,4,6-Gal*p*	3,4,6-linked galactose pyranose
2,3,4,6-Gal*p*	2,3,4,6-linked galactose pyranose
2,4-Glc*p*+2,4-Gal*p*	2,4-linked glucose pyranose and 2,4-linked galactose pyranose
t-Glc*p*	Terminal glucose pyranose

3-Glc*p*	3-linked glucose pyranose
4-Glc*p*	4-linked glucose pyranose
6-Glc*p*	6-linked glucose pyranose
2,3-Glc*p*	2,3-linked glucose pyranose
3,4-Glc*p*	3,4-linked glucose pyranose
3,6-Glc*p*	3,6-linked glucose pyranose
4,6-Glc*p*	4,6-linked glucose pyranose
2,3,6-Glc*p*	2,3,6-linked glucose pyranose
2,4,6-Glc*p*	2,4,6-linked glucose pyranose
3,4,6-Glc*p*	3,4,6-linked glucose pyranose
2,3,4,6-Glc*p*	2,3,4,6-linked glucose pyranose
t-Man*p*	Terminal mannose pyranose
2-Man*p*	2-linked mannose pyranose
3-Man*p*	3-linked mannose pyranose
4-Man*p*	4-linked mannose pyranose
2,3-Man*p*	2,3-linked mannose pyranose
2,4-Man*p*	2,4-linked mannose pyranose
2,6-Man*p*	2,6-linked mannose pyranose
3,4-Man*p*	3,4-linked mannose pyranose
3,6-Man*p*	3,6-linked mannose pyranose
4,6-Man*p*	4,6-linked mannose pyranose
2,3,6-Man*p*	2,3,6-linked mannose pyranose
2,4,6-Man*p*	2,4,6-linked mannose pyranose
3,4,6-Man*p*	3,4,6-linked mannose pyranose
2,3,4,6-Man*p*	2,3,4,6-linked mannose pyranose
t-Rha*p*	Terminal rhamnose pyranose
2-Rha*p*	2-linked rhamnose pyranose
3-Rha*p*	3-linked rhamnose pyranose

4-Rha*p*	4-linked rhamnose pyranose
2,3-Rha*p*	2,3-linked rhamnose pyranose
2,4-Rha*p*	2,4-linked rhamnose pyranose
3,4-Rha*p*	3,4-linked rhamnose pyranose
2,3,4-Rha*p*	2,3,4-linked rhamnose pyranose
t-Xyl*p*	Terminal xylose pyranose
2-Xyl*p*	2-linked xylose pyranose
3-Xyl*p*	3-linked xylose pyranose
4-Xyl*p*	4-linked xylose pyranose
2,4-Xyl*p*	2,4-linked xylose pyranose
3,4-Xyl*p*	3,4-linked xylose pyranose
2,3,4-Xyl*p*	2,3,4-linked xylose pyranose
t-Gal*p*A	Terminal galacturonic acid pyranose
2,4-Glc*p*A+2,4-Gal*p*A	2,4-linked glucuronic acid pyranose and 2,4-linked galacturonic pyranose
t-Glc*p*A	Terminal glucuronic acid pyranose
3-Glc*p*A	3-linked glucuronic acid pyranose
4-Glc*p*A	4-linked glucuronic acid pyranose
t-Gul*p*A	Terminal guluronic acid pyranose
4-Gul*p*A	4-linked glucuronic acid pyranose
t-Man*p*A	Terminal mannuronic acid pyranose
4-Man*p*A	4-linked mannuronic acid pyranose

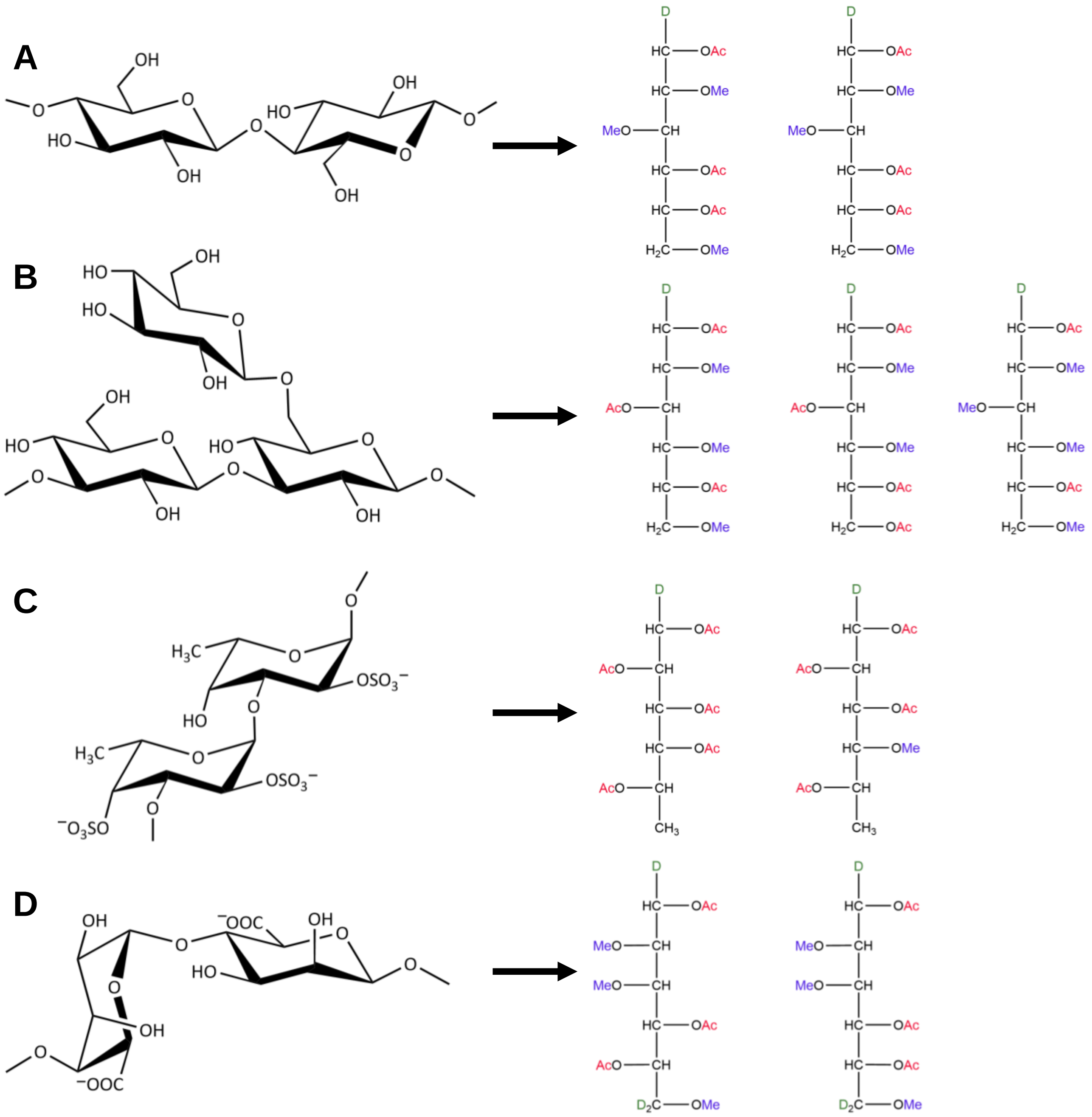

Scheme S3. Diagram showing the PMAAs generated from: (**A**) cellulose, (**B**) laminarin, (**C**) a sulfated fucan, and (**D**) alginate.
